# Informal state influence in international organizations: examining the link between executive head nationality and earmarked funding

**DOI:** 10.1177/13540661251355045

**Published:** 2025-07-23

**Authors:** Giuseppe Zaccaria, Bernhard Reinsberg

**Affiliations:** University of Glasgow, UK

**Keywords:** International organizations, principal-agent theory, earmarked funding, multilateral system, IO bureaucracies

## Abstract

This article examines whether and how donor states engage differentially with earmarked funding within the multilateral system when their nationals hold executive office in international organizations (IOs). The article relies on recent scholarship on multilateral finance and IO bureaucracies to identify executive representation and earmarked funding as tools that states may use for exerting influence within IOs. Based on that, the article expects donors to both allocate smaller earmarked contributions for individual IO activities and to earmark their contributions less stringently when their nationals run IOs, while increasing the total volume of earmarked funding they provide to IOs. Using data from over 110,000 multilateral activities implemented by 49 IOs (1990–2020), our analyses suggest that, when their nationals run IO-executive offices, donors increase the overall (yearly) volume of earmarked funding they provide to IOs while decreasing the size of their individual earmarked contributions for IO activities, and their contributions tend to be also more stringently earmarked. The findings also suggest that donor considerations towards earmarked funding may be shaped by the tenure of executive heads. These findings have important implications for IO scholars, policymakers and stakeholders.

## Introduction

Powerful states famously invest in placing their nationals at leadership positions in IOs. The president of the World Bank has always been a US citizen (Weaver, 2007), while the International Monetary Fund (IMF) is always run by a European managing director. The US stands out in this regard, having put great effort in the leadership selection processes of multilaterals, such as the United Nations Children’s Fund, the International Organization for Migration, the office of the UN Secretary-General, the head of the Organization for the Prohibition of Chemical Weapons (Hall and Woods, 2018; World Economic Forum, 2014) and even the World Food Programme.^1^

Executive heads have substantial influence over the political system of IOs (Biermann and Siebenhüner, 2009; Hall and Woods, 2018; Yi-chong and Weller, 2018). They oversee key functions, set agendas, initiate policies, represent and legitimatize their institutions in external engagements and serve as change agents (Cox, 1969; Hall and Woods, 2018; Kille and Scully, 2003; Schechter, 1987, 2012; Schroeder, 2014; Von Billerbeck, 2022; Yi-chong and Weller, 2018). It should therefore be unsurprising that powerful states vie to place their nationals at the helm of IOs and to rely on them for aligning IOs decisions and outcomes with their preferences. Despite that conventional wisdom, systematic large-N analysis of variation in states’ behaviour within IOs resulting from their control of executive offices remains limited.

Towards filling that gap, this article assesses the relationship between executive heads’ nationality and the allocation of donor states’ earmarked contributions, a good proxy for states’ efforts at exerting influence over an institution beyond traditional (collective) processes. The ability to earmark voluntary contributions provides donors with added leverage over the operations and goals of IOs. Unsurprisingly, earmarked funding is seen as increasing donor control and preference alignment at the expense of the autonomy, multilateral nature and performance of IOs (Eichenauer and Reinsberg, 2017; Graham, 2023; Heinzel et al., 2023; Reinsberg, 2017).

On that backdrop, this article expects donors’ engagement with earmarked funding to vary according to the nationality and tenure of IO-executive heads. The article focuses on changes in the size and overall volume of donor-earmarked contributions as well as the degree of stringency with which these are earmarked. Size of contribution denotes the amount of earmarked funding provided by a donor for an individual IO activity (activity level), while volume denotes the total amount of donor-earmarked funding provided to an IO in a year (IO-donor-year level). Stringency denotes the degree to which a donor specifies the purpose of its contributions (activity level). For example, a contribution earmarked for a specific project theme and for a specific country is more (stringently) earmarked than a contribution specified for a broad regional programme.

The article presumes that the control of executive offices provides donors with enough institutional leverage to ensure that policies and decisions are aligned with their preferences. This theoretical conjecture, derived from the insights of previous studies, is key to our argument. Based on that conjecture, we theorize that donors decrease the size of their earmarked contributions for IO activities and earmark their contributions less stringently, when their national runs IOs. At the same time, we expect donors to provide larger overall volumes of earmarked funding to IOs during the executive tenure of their nationals, following the logic of trust and donor generosity, which should incentivize them towards supporting IO mandates and operations led by their nationals.

Moreover, as part of the selection process of IO executives, donors likely make promises to curry favour with members and secure votes for their candidates. In a bid to keep their promises and align IO operations with the preferences of those members, donors are likely to increase the size of their contributions for IO activities, boost the volume of their overall funding and increase the stringency with which they earmark their contributions when their nationals *begin* their executive tenure. As the executive tenure of their national approaches its *end*, donors may behave similarly to ensure that future IO operations remain aligned with their preferences, or to align IO operations with the preferences of other members and secure their future vote for the (re-)election of their candidates.

To test its expectations, the article relies on data on executive heads and earmarked funding for over 110,000 aid activities implemented by 49 IOs (1990–2020). While a causal link between the control of executive offices, earmarked funding and donor goals is outside the scope of this article, nevertheless the results suggest that donors’ differential engagement with earmarked funding is correlated with variation in executive representation at IOs. The results indicate that donors generally provide smaller-sized earmarked contributions for individual IO activities when their nationals run IO-executive offices, but these contributions tend to be more stringently earmarked, as compared to when their nationals are not in office. Moreover, the overall (yearly) volume of earmarked funding provided by donors indeed increases when their nationals are in executive office. Finally, donors earmark their contributions more stringently towards the end of their nationals’ executive tenure.

This article contributes to research on informal state influence in global governance (Kleine, 2013; McLean, 2017; Stone, 2004, 2011; Urpelainen, 2012; Vreeland, 2019). The article builds on determinants of multilateral funding (Dreher et al., 2009; Kersting and Kilby, 2016; Kilby, 2009; Vreeland and Dreher, 2012). The article adds to that literature by revealing how states’ behaviour within IOs can vary according to staffing dynamics – and hence potentially the degree of control they may exert – within the executive level of IOs. The article also helps ascertain the Generalizability of previous findings on the dynamics of member states’ bureaucratic influence in IOs (Gehring and Schneider, 2018; Gui, 2024; Kaja and Werker, 2010; Novosad and Werker, 2019) by systematically examining such patterns within a large sample.

Moreover, studies have highlighted a rise in IO authority in recent decades, suggesting that international bureaucracies are increasingly effective at pushing their preferences with some degree of independence from member states (Bauer and Ege, 2016; Johnson, 2013, 2014; Zürn et al., 2021). The findings encourage further scholarly research on alternative channels of influence employed by principals in the face of such rise in IO authority. Earmarked funding, by empowering individual principals, establishes additional control mechanisms beyond collective governance mechanisms. As earmarked funds have become increasingly crucial to IO resources (Heinzel et al., 2023), the findings suggest that the pathways for influence they offer require also a renewed attention to the problem of IO autonomy.

Moreover, the article speaks directly to the scholarship on earmarked funding in IOs by focusing on its institutional drivers. Extant studies often identify earmarked funding as a tool employed by states for shaping the goals and operations of IOs and to align these with their domestic priorities (Graham, 2017b; Reinsberg, 2017). Previous studies have shown how states’ choice between multilateral, earmarked and bilateral funding channels is determined by IO governance rules, preference-heterogeneity among states and domestic concerns regarding aid effectiveness (Bayram and Graham, 2017; Eichenauer and Hug, 2018; Graham and Serdaru, 2020). This article advances that debate by revealing the relevance of institutional factors to donors’ funding behaviour, notably executive representation in IOs.

## Informal state influence, executive heads and earmarked funding in IOs

The principal-agent framework expects states to be intrinsically motivated to increase their informal influence within IOs in a bid to align policies with their preferences (Abbott and Snidal, 1998; Hawkins et al., 2006; Stone, 2011; Urpelainen, 2012). Towards that, states invest into geographic representation in IO bureaucracies (Elsig and Pollack, 2014; Kleine, 2013), as like-minded co-nationals are expected to facilitate preference alignment and enhance their informal influence (Badache, 2020; Heinzel, 2022c; Novosad and Werker, 2019; Parízek, 2017). As previous studies show, influence is reflected in the over-representation of nationals from powerful states in IO bureaucracies (Eckhard and Steinebach, 2021; Novosad and Werker, 2019). States sometimes even focus on specific IO divisions, thus inducing the creation of national fiefdoms (Kleine, 2013). Bureaucratic staffing is a useful avenue for informal influence in IOs, particularly regarding executive offices.

States’ incentives to control executive offices is rather intuitive, especially if one is to consider IO bureaucracies as enjoying authority (Barnett and Finnemore, 2004; Bauer and Ege, 2014; Biermann and Siebenhüner, 2009; Dijkstra et al., 2022; Johnson, 2013; Schuette, 2021; Vaubel et al., 2007). In IOs’ complex and multilayered political system, a hierarchical bureaucratic structure projects authority from the executive level to lower echelons (Hall and Woods, 2018; Yi-chong and Weller, 2018). This puts executive heads in a powerful institutional position, from which they oversee and intervene in key processes such as budgeting, agenda-setting and initiating policies; they also help legitimatize and represent their institutions in external engagements and, sometimes, even serve as change agents (Cox, 1969; Hall and Woods, 2018; Kille and Scully, 2003; Patz and Goetz, 2019; Schechter, 1987, 2012; Schroeder, 2014; Von Billerbeck, 2022; Yi-chong and Weller, 2018). Key institutional actors are shown to exploit opportunity structures and their formal competences to engage with issues, steer the direction of their IOs and collude with member states (Dijkstra, 2017; Dijkstra et al., 2022).

The degree of authority that executive heads hold varies across IOs. Some enjoy significant institutional powers, such as agenda-setting and policy-proposal authority, which help them directly shape decision-making processes in IOs (Hall and Woods, 2018; Hooghe and Marks, 2015; Stone, 2011). Others hold less authoritative roles, acting mainly as head managers of IO bureaucracies, with fewer opportunity structures to influence governance and outcomes (Dijkstra et al., 2022; Elsig, 2010). Although states may be more incentivized to place their nationals in IOs with more formal authority (Heinzel, 2022c), it is reasonable to suggest that they will nevertheless still gain from controlling executive offices even in IOs with less formal authority. That is because executive heads have *informal* authority.

Executive heads often take on informal roles by serving as diplomatic facilitators and channels of communication between states and their home countries (Johns, 2007; Mathiason, 2007, 2018; Mele et al., 2016; Spies, 2015). IO executives are not dissimilar to their counterparts in public agencies, who are often seen as leaning more towards the political side in the grey zone between politics and administration (Christensen, 1991). States are keenly aware of these opportunity structures. When designing new IOs, powerful states invest into maximizing the autonomy of their bureaucracies to ensure that channels of informal influence can be later employed in their interest (Manulak, 2017). As the negotiations over the design of the United Nations Environment Programme (UNEP) illustrate, less powerful states seek to minimize the autonomy of new IOs, as they predict themselves holding less informal influence over their bureaucracies (Manulak, 2017).

It is therefore unsurprising that powerful states vie to place their nationals at the helm of IOs. Candidates for the job are regularly picked from within the national policy networks, often holding prior experience in ministries and governments (Heinzel, 2022c). They likely hold strong national allegiances and intrinsic motivations (i.e. career plans) to represent their countries’ interests (Hall and Woods, 2018; Heinzel, 2022c; Kahler, 2001). They often owe their role to related lobbying efforts by their home countries’ governments and take up powerful positions in their countries upon the end of their executive terms in IOs, thus making them likely to carefully approach their executive role with their future prospects in mind (Gehring and Schneider, 2018; Vaubel et al., 2012).

Several high-profile cases illustrate this. At the United Nations, the French head of the Oil-for-Food programme in Iraq, Benon Savan, prioritized the allocation of oil towards French companies and individuals, while the Assistant Secretary-General from Myanmar, Tun Myat, prioritized requests from his co-nationals, thus facilitating the award of contracts to companies from Myanmar (Novosad and Werker, 2019). Meanwhile, the American assistant director of the United Nations Special Commission in Iraq (and former Deputy Assistant Secretary in the U.S. Department of State), Charles Duelfer, was accused of acting as a key intermediary in providing espionage coverage for Central Intelligence Agency agents through the agency (Novosad and Werker, 2019).

We presume that executive heads likely provide their home countries a useful channel through which to influence institutional processes. We also acknowledge that executive heads face pressures that promote their impartiality and neutrality (Verbeek, 2009). However, as the anecdotal examples of executive bias and scholarly insights on member states’ strategic efforts towards representation in IO bureaucracies suggest, pressures for impartiality may not always curtail collusion at the executive level. Moreover, our theoretical expectations do not fundamentally contrast those of studies that highlight the motivations of IO bureaucracies towards establishing an image of neutrality and impartiality. Indeed, we expect both pressures promoting impartiality and bias to occur concurrently within the political system of IOs.^2^

In the absence of executive control, member states may explore other avenues of influence within IOs. More specifically, this article expects variation in states’ executive representation in IOs to reflect on their earmarked funding behaviour.

### Linking executive features to variation in earmarked funding in IOs

IO funding modalities fall broadly within two categories: assessed and voluntary contributions. Assessed contributions essentially correspond to membership fees, whereby each member state pays a sum into the budget of an IO determined by the establishing treaty. Voluntary contributions, in contrast, are determined by individual member states and can be unearmarked or earmarked. Unearmarked contributions are flexible and used by IO bureaucracies with discretion. Earmarked contributions, in contrast, denote an increasingly common funding modality whereby donor states fund IO activities through voluntary contributions with strings attached. These contributions can be earmarked on various dimensions, such as for specific themes, recipient countries and institutional divisions (Graham, 2017a, 2017b; Heinzel et al., 2023).

While earmarked funding may help expand IOs’ resource pool (Eichenauer and Reinsberg, 2017; Reinsberg et al., 2015, 2024), it also offers donor states more leverage over IO operations at the expense of IO discretion, autonomy and performance (Heinzel et al., 2023; Schmid et al., 2021). Earmarked funding has been shown to negatively affect performance in IOs as compared to core funding (Reinsberg et al., 2024) and to have detrimental effects on the conditions that allow IOs to effectively plan and manage operations, liaise with partners, monitor results and promote institutional learning (Reinsberg and Siauwijaya, 2023). This may be due to strict monitoring requirements that come with earmarking and the consequent additional administrative burden shouldered by IO staff (Gaus et al., 2020; Heinzel and Reinsberg, 2024; Heinzel et al., 2023; Reinsberg, 2023; Reinsberg and Siauwijaya, 2023).^3^ Concerns over earmarked funding are reflected in an Organisation for Economic Co-operation and Development (OECD) report which identifies the rise of transaction-heavy ‘piecemeal’ funding – and the resulting low degree of predictability in financing for IOs – as an increasingly significant challenge to the ability of IOs in delivering on their mandates (OECD, 2018).

Despite concerns and potential pitfalls associated with earmarked funding, states have increasingly opted for earmarking their voluntary contributions. In global health cooperation, for example, there is evidence of a marked rise in the use of earmarked funding, promulgated through donor-financed vertical funds with narrow problem-based goals (Sridhar and Woods, 2013). Extant studies offer various explanations for states’ motivations in engaging with earmarked funding. One perspective focuses on the political economy of donor states and views governments as agents and the electorate as principals, explaining states’ reliance on earmarked funding as aimed at enhancing their ability to scrutinize IOs and address domestic accountability (Eichenauer and Reinsberg, 2017). Another perspective views IOs as agents and states as principals, explaining earmarking as a tool employed by states to enhance their ability at shaping the goals and operational scope of IOs and to align these with their priorities (Reinsberg, 2017).

For example, some studies suggest states’ choice between multilateral, earmarked and bilateral channels of funding to be determined by IO governance rules, the degree of preference-heterogeneity among states, as well as domestic concerns and concerns regarding aid effectiveness (Eichenauer and Hug, 2018; Graham and Serdaru, 2020), particularly when states’ priorities do not align well with those of other member states in IO governance boards (Bayram and Graham, 2017). Earmarked funding often foregoes voting and power dynamics in collective governance organs. It may allow states to substitute voting dynamics when it comes to resource allocation, especially where states have equal influence in governing bodies (Graham and Serdaru, 2020). Thus, earmarked funding can be understood as a mechanism for informal state influence, not dissimilar to other mechanisms employed in respect to formal processes in governance boards (Kilby, 2013) – even though decisions of trust fund support at multilaterals such as the World Bank can be fairly formalized (Reinsberg and Steinwand, 2025).

States are also shown to rely on earmarked funding as a strategic choice for expanding the scope of an IO beyond its mandate (Graham, 2017b). Other studies suggest that some states rely on earmarked funding for projects in high-risk and low-governance countries (Eichenauer and Reinsberg, 2017). These factors may combine in motivating states’ reliance on earmarked funding. Nevertheless, the overarching view in the literature is that donor states allocate earmarked funding strategically and according to their interests.

As such, this article expects donor states to rely on earmarked funding arrangements to push their preferences and align IO operations with their priorities differentially according to whether their nationals hold executive office. When executive offices are staffed by their nationals, donor states are expected to provide smaller contributions to IO activities. This is because, following the extant literature on IO executives and bureaucratic dynamics, the powerful role of executive heads is expected to provide donors with the reassurance that IO activities will be, to some degree, aligned with their preferences. Larger-sized contributions may be more effective at shaping individual IO activities, as donor requirements may carry a heavier weight on substantive goals and operational priorities of activities funded more generously through voluntary donor contributions. Thus, we expect donors to allocate, on average, larger-sized earmarked contributions for individual IO activities when they lack executive representation.

At the same time, however, the presence of donor nationals at the helm of an IO may encourage donors to provide larger overall (yearly) volumes of earmarked funding to an IO, given the increased trust that they may feel towards the IO.^4^ Moreover, donors with executive representation may be motivated to support the campaigning of their executive nationals by providing additional resources to their institution. This may occur even if donors’ individual contributions to specific IO activities shrink in size, as donors may spread their funding across a broader range of IO activities. Hence, we assume the size of earmarked contributions to IO activities to reflect how invested donors are in *shaping* IO activities, while overall volumes of earmarked funding reflect how generous donors are towards *supporting* IOs.

Donors are also expected to earmark their contributions less stringently when they feel that IO operations and programmes are more aligned with their preferences, i.e. when donor states’ nationals lead the executive office in IOs. Earmarking stringency denotes how strictly a voluntary contribution is earmarked. For example, voluntary contributions earmarked by a donor for a specific project theme (e.g. rural health infrastructure) in a specific country are considered more stringently earmarked than contributions earmarked for broad IO programmes (e.g. food security) focusing on a wider region. The stringency of earmarking can be a powerful tool for donors to limit the discretion of IO officials and to bind operations to their preferences. Thus, we expect executive nationality to be associated with variation on all three dimensions of earmarked funding, hence the following set of hypotheses:

*Hypothesis 1 (H1). Donors provide smaller-sized contributions for individual multilateral activities implemented by IOs that are led by their nationals.*
*Hypothesis 2 (H2). Donors provide larger overall (yearly) volumes of earmarked contributions to IOs that are led by their nationals.*
*Hypothesis 3 (H3). Donors earmark less stringently their contributions to multilateral activities implemented by IOs that are led by their nationals.*

Donor considerations regarding earmarked funding may vary not only based on whether their nationals are in executive positions at IOs but also based on the tenure of these officials.^5^ First, earmarked funding may provide donors with a tool for currying favour with other member states. By promising larger earmarked contributions in support of IO activities that align with the interests of specific member states, donor states may secure votes for their proposals and, importantly, for their candidates to executive office. This is expected to be associated with a noticeable increase in the size of individual contributions to IO activities at the onset of the executive tenure of donor nationals in IOs, as promises begin to materialize. We expect the overall (yearly) volume of earmarked funding provided by donors to an IO to also increase at the start of the tenure of their executive heads, as part of their supporting efforts for the campaigns and agendas of their executive nationals. This should also affect the degree of stringency with which donors earmark their voluntary contributions, with more earmarking applied to specific programmes and for specific geographic locations that may appease other members and secure their potential vote for donors’ executive candidates. Thus, the following hypotheses:

*Hypothesis 4 (H4). Donors provide larger-sized contributions for individual multilateral activities implemented by IOs at the start of the executive tenure of their nationals.*
*Hypothesis 5 (H5). Donors provide larger overall (yearly) volumes of earmarked contributions to IOs at the start of the executive tenure of their nationals.*
*Hypothesis 6 (H6). Donors earmark more stringently their contributions to multilateral activities implemented by IOs at the start of the executive tenure of their nationals.*

Donor states may also engage more proactively with earmarked funding towards the end of the executive tenure of their nationals. Assuming that donor states value having their nationals hold such positions, they are expected to provide, on average, larger earmarked contributions for individual IO activities to gain the support of other members for their incumbent as their executive term comes to end, in order to ensure their continued tenure (Reinsberg, 2019). They may also provide larger overall volumes of earmarked funding to boost broader IO programmes during the executive tenure of their nationals. At the same time, as earmarking contributions provide donors with control over the allocation of IO resources for specific programmatic and geographic priorities, donor states may rely on earmarking more intensely in a bid to ensure that future IO operations remain aligned with their preferences even if their nationals are not re-elected. Thus, the following hypotheses:

*Hypothesis 7 (H7). Donors provide larger-sized contributions for individual multilateral activities implemented by IOs at the end of the executive tenure of their nationals.*
*Hypothesis 8 (H8). Donors provide larger overall (yearly) volumes of earmarked contributions to IOs at the end of the executive tenure of their nationals.*
*Hypothesis 9 (H9). Donors earmark more stringently their contributions to multilateral activities implemented by IOs at the end of the executive tenure of their nationals.*

## Research design

To test its expectations, the article draws on a dataset covering over 110,000 activities implemented by 49 IOs in 174 recipient countries, funded through earmarked contributions by 41 donor states between 1990 and 2020. The analysis first examines the effect of co-nationality of executive heads on the size of donors’ earmarked contributions for individual IO activities and the overall (yearly) volume of earmarked funding they provide to IOs. Next, it examines the effect of co-nationality of executive heads on the degree of stringency attached by donors to their earmarked contributions for individual IO activities. Finally, the analysis focuses on the interaction effect between IO-executive heads’ tenure and nationality on the variation in size and earmarking stringency of individual donor contributions, as well as on the overall (yearly) volume of donor-earmarked funding. The analyses rely on Ordinary Least Squares (OLS) regressions with IO, donor, year, recipient and sector fixed effects to control for unobserved differences at various levels, as well as a wide range of control variables to mitigate omitted-variable bias.^6^

### Dependent variables

The analyses focus on three main dependent variables: the size of earmarked contributions provided by donors to individual IO activities, the overall (yearly) volume of earmarked funding by donors to IOs and the earmarking stringency index of donor contributions to individual IO activities. Data for these are taken from the Earmarked Funding Dataset (Eichenauer and Reinsberg, 2017; Reinsberg et al., 2024).

The first dependent variable measures the size of the earmarked contribution provided by donors to individual IO-implemented activities (*Size*). This is at the activity level and measured in million USD (logged for the analysis). As such, it does not measure the overall volume of earmarked funding provided by a donor each year, but only the size of each individual donor-earmarked contribution provided for IO activities available in the Earmarked Funding Dataset. The second dependent variable measures the yearly volume of overall earmarked funding provided by donors to IOs (*Volume*). This is at the IO-donor-year level and is measured in millions USD (logged for the analysis). It measures the sum of all earmarked contributions provided by a donor to IO activities each year. The third dependent variable measures the degree of stringency of earmarked contributions by donors for IO activities (*Stringency*). Stringency at the activity level is reflected in various dimensions on which donors’ contributions are earmarked, including geographical, thematic and institutional. Each dimension is scored depending on the degree of stringency attributed.

Along the geographical dimension, a contribution can be unearmarked (score of 0), earmarked for an activity implemented in a specific region (1) or a specific country (2). Along the thematic dimension, a contribution can be unearmarked (0), earmarked for an activity within a broad IO programme (1), or a specific theme (2). Along the institutional dimension, a contribution can be unearmarked (0), earmarked for an IO division (1), for a specific actor within the IO (2), or require the secondment of donor staff to the IO (3). In addition, some donors earmark their contributions for several institutional dimensions concurrently, such as for a division or specific actor in addition to staff secondment (Reinsberg et al., 2024). A stringency index is then constructed for each dimension by dividing the score by the range in that dimension. For example, for the geographical dimension, a contribution earmarked regionally would score 1. The score is then divided by 2 (as that dimension has a stringency index range of 0–2), and the resulting value (1/2) indicates the contribution’s overall geographic earmarking. The overall stringency index is then constructed by summing up the scores for each earmarking dimension (range of 0–3).

Additional analyses take a more granular view on earmarking stringency at the activity level. For this, earmarking is left disaggregated along the three dimensions of thematic (*Thematic stringency*), geographic (*Geographic stringency*) and institutional (*Institutional stringency*) earmarking explained earlier. The analysis then examines how executive nationality is associated with variation in stringency within each of those dimensions. Table 1 presents the descriptive statistics of the data.

**Table 1.** Descriptive statistics.

Variable	*N*	Mean	SD	Min	Max
Executive co-nationality	110,671	0.17	0.378	0	1
Executive tenure start	110,671	0.15	3.58	0	1
Executive tenure end	110,671	0.22	0.41	0	1
Size of donor-earmarked contribution to IO activity	110,671	2.38	9.55	−2.17	905
Earmarking stringency of contribution	110,671	1.41	0.48	0	2.80
Thematic earmarking stringency	110,671	0.59	0.32	0	1
Geographic earmarking stringency	110,671	0.78	0.37	0	1
Institutional earmarking stringency	110,671	0.03	0.14	0	0.80
Overall volume of donor-earmarked funding to IO (yearly)	8,865	20.3	109	−0.44	2,980
Number of donor-earmarked activities at IO (yearly)	8,865	12.5	39	1	1,020
Donor all multilateral funding (yearly, lag)	8,865	1,480	1,530	0	6,860
Donor all earmarked funding (yearly, lag)	8,865	749	1,230	0	7,350
Donor all bilateral funding (yearly, lag)	8,865	4,280	6,790	12.7	36,500
Donor GDPPC (yearly, lag)	8,865	42.2	10.9	18.9	92.40
Donor democracy (yearly)	8,865	0.8	0.11	0.04	0.89
Donor ideal-point (yearly)	8,865	1.12	0.51	−1.25	3.04
Ideal-point distance (yearly)	8,865	1.12	0.77	0	4.23

### Independent variable

For the independent variable, a dichotomous indicator is constructed (*Executive co-nationality*) for whether the executive head of an IO matches the nationality of the donor. At the IO activity level, this indicates that an activity which received an earmarked contribution by a donor was implemented by an IO led by that donor’s national. For example, when an individual IO activity is funded through an earmarked contribution by country A, and the nationality of the IO-executive head is also from country A, then the independent variable is scored 1. Where this is not the case, the score is 0. A similar logic is applied for constructing the independent variable at the IO-donor-year level. To collect data on the nationality of IO-executive heads, the article relies on official IO sources and data from the Biographical Dictionary of Secretaries-General of International Organizations project (Reinalda et al., 2017).^7^ In total, the dataset covers executive heads from 87 nationalities who were in office in the period 1990–2020 in the 49 IOs included in the sample.

### Control variables

The analyses rely on a wide range of control variables at the donor and IO-executive levels to mitigate omitted-variable bias. These include the yearly number of projects funded through earmarking by a given donor in a given IO (*Number of donor-funded projects in IO*), the yearly volume of earmarked funding provided by a given donor to a given IO (*Donor earmarked commitments to IO*), the yearly volume of all earmarked funding provided by a given donor to all IOs (*Donor total earmarked commitments*), the yearly volume of all multilateral funding provided by a given donor (*Donor total multilateral commitments*) and the yearly volume of all bilateral funding provided by a given donor (*Donor total bilateral commitments*). Donors with higher previous multilateral funding, bilateral funding and earmarked funding in IOs may be more likely to engage in earmarked funding in general. The measure for the total number of projects funded by donors is also included as a wider project portfolio may affect earmarked funding arrangements made by donors for IO activities. Data for these variables are sourced from the Earmarked Funding Dataset (Eichenauer and Reinsberg, 2017; Reinsberg et al., 2024).

The analyses also control for donors’ GDP per capita (*Donor GDP per capita*), level of democracy (*Donor democracy*), political ideal-point in relation to the US-led liberal order based on United Nations General Assembly (UNGA) voting and their ideal-point distance from the country of origin of IO-executive head (*Donor ideal-point* and *ideal-point distance*, respectively). Donors with more economic prowess may engage more generously with earmarked funding. More democratic donor countries and those more supportive of the liberal multilateral order may be more willing to rely on voluntary funding to support multilateral institutions. A wider distance between the ideal-points of donor countries and IO-executive heads’ countries of origin may also affect the degree of trust that donors feel towards an IO. Data for GDP per capita and democratic features are sourced from the ‘Varieties of Democracy’ (*V-Dem*) project (Coppedge et al., 2022). Data on UNGA voting are sourced from the United Nations General Assembly voting alignment data (Bailey et al., 2017).

In addition, an indicator is constructed (based on the data used for the independent variable) measuring the years in which the executive head has been in office. Based on this, two new variables are constructed. These indicate whether the executive head has just entered office (*Tenure start*) and whether they are in the final year of their tenure (*Tenure end*). These are included to account for the possibility that donors may tailor their earmarking portfolios according to the tenure of executive heads. The interaction between executive heads’ tenure and executive nationality is used to test the expectations in H4–H9.

## Results

Table 2 examines the effect of IO heads’ co-nationality on the size of earmarked commitments by donors to individual IO activities.

**Table 2.** IO-executive co-nationality and size of donor-earmarked commitments (activity level).

	1 Size	2 Size	3 Size	4 Size
Co-nationality	−0.827*** (0.033)	−0.825*** (0.033)	−0.342*** (0.030)	−0.506*** (0.048)
Tenure (start)		−0.011 (0.021)	−0.010 (0.019)	−0.013 (0.020)
Tenure (end)		−0.033 (0.020)	−0.040* (0.019)	−0.039* (0.019)
Number of donor-funded projects in IO (yearly, log)			−0.966*** (0.009)	−0.974*** (0.010)
Donor earmarked commitments to IO (yearly, log)			0.801*** (0.007)	0.800*** (0.007)
Donor total earmarked commitments (yearly, log, lagged)			0.078*** (0.009)	0.078*** (0.009)
Donor total multilateral commitments (yearly, log, lagged)			−0.006 (0.016)	−0.006 (0.016)
Donor total bilateral commitments (yearly, log, lagged)			−0.074** (0.026)	−0.061* (0.026)
Donor GDP per capita (log, lagged)			1.062*** (0.165)	0.978*** (0.166)
Donor democracy				−0.820*** (0.245)
Donor ideal-point				0.125* (0.052)
Ideal-point distance				−0.078*** (0.015)
Observations	75,793	75,793	74,756	74,351
R^2^	0.281	0.281	0.399	0.400
Fixed effects: IO	Yes	Yes	Yes	Yes
Fixed effects: Donor	Yes	Yes	Yes	Yes
Fixed effects: Recipient	Yes	Yes	Yes	Yes
Fixed effects: Year	Yes	Yes	Yes	Yes
Fixed effects: Sector	Yes	Yes	Yes	Yes

Clustered standard errors in parentheses.

+ *p* < 0.1, *p < 0.05, **p < 0.01, ***p < 0.001.

Model 1 includes the independent variable as well as IO, donor, recipient, sector and year fixed effects for estimating its effect on the dependent variable. Model 2 includes variables indicating the executive tenure (start and end). Model 3 includes the donor-level control variables relevant to its multilateral funding portfolio. Model 4 includes all control variables. The results in Table 2 align with expectations: variation in the size of donor-earmarked commitments to individual IO activities is associated with the nationality of IO heads. The coefficient of the independent variable is negative and statistically significant (*p* < 0.001) in all models. Thus, the results suggest that when the nationality of an IO’s executive head is the same as that of a donor, that donor is significantly likely to provide smaller earmarked contributions for IO activities. More specifically, the presence of donor nationals in the executive office of an IO is associated, on average, with an approximate decrease of approximately 40% in the size of individual contributions, based on the exponentiated value of the coefficient in Model 4. This implies a decrease of about USD 0.92 million per contribution at the mean. This is a sizable decrease as the average size (mean) across the contributions in the sample is USD 2.3 million.

Table 3 examines the effect of co-nationality on the overall volume of earmarked funding provided by a donor to an IO in each given year. Model 5 includes only the independent variable as well as IO, donor and year fixed effects. Subsequent models add substantive control variables. The results in Table 3 align with our expectations: co-nationality at the IO executive is positively associated with the overall (yearly) volume of earmarked funding. The presence of donor nationals in the executive office of an IO is associated, on average, with an approximate increase of 0.37 points (*p* < 0.001) in the dependent variable (based on Model 8). This translates into an average increase of 46%, or USD 9.2 million at the mean (USD 20 million) in overall (yearly) volume of voluntary earmarked funding provided by donors to IOs each year.

**Table 3.** IO head co-nationality and overall (yearly) volume of earmarked funding (IO-donor-year level).

	5 Volume	6 Volume	7 Volume	8 Volume
Co-nationality	1.026*** (0.134)	1.026*** (0.134)	0.489*** (0.095)	0.379*** (0.108)
Tenure (start)		0.038 (0.057)	0.020 (0.048)	0.023 (0.048)
Tenure (end)		0.022 (0.056)	0.027 (0.048)	0.027 (0.048)
Number of donor-funded projects in IO (yearly, log)			0.979*** (0.020)	0.978*** (0.020)
Donor total earmarked commitments (yearly, log, lagged)			0.128*** (0.024)	0.130*** (0.024)
Donor total multilateral commitments (yearly, log, lagged)			0.029 (0.046)	0.004 (0.048)
Donor total bilateral commitments (yearly, log, lagged)			0.107 ^+^ (0.062)	0.135* (0.062)
Donor GDP per capita (log, lagged)			1.553*** (0.366)	1.629*** (0.368)
Donor democracy				−0.748 (0.651)
Donor ideal-point				−0.074 (0.161)
Ideal-point distance				−0.079* (0.035)
Observations	7767	7767	7407	7326
R^2^	0.527	0.527	0.643	0.646
Fixed effects: IO	Yes	Yes	Yes	Yes
Fixed effects: Donor	Yes	Yes	Yes	Yes
Fixed effects: Year	Yes	Yes	Yes	Yes

Clustered standard errors in parentheses.

+ *p* < 0.1, *p < 0.05, **p < 0.01, ***p < 0.001.

Table 4 examines the effect of IO heads’ co-nationality on the stringency with which donors earmark their contributions for individual IO activities. Model 9 only includes IO, donor, year, recipient and sector fixed effects. Model 10 further includes variables that identify the start and end of the tenure of executive heads. Model 11 additionally includes controls for donor-level control variables relevant to its funding portfolio. Model 12 controls for democratic governance, donor-IO head ideal-point distances and donors’ ideal-points distance. The coefficient of the independent variable is positive and statistically significant (*p* < 0.001) in all models: when IOs are headed by the national of a donor country, the IO activities that are funded through voluntary contributions by that donor are more likely to be earmarked more stringently. Based on Model 12, the presence of donor nationals in the executive office is associated, on average, with an increase of about 0.04 points in the stringency (range 0–3) with which that donor earmarks its contributions to the IO activity. Although this contrasts the expectation in H3, the results warrant caution in interpretation. The coefficient in Model 12 is very small and does not effectively translate to a meaningful change in sub-dimensional earmarking. In other words, based on the results in Model 12 it seems unlikely that executive head co-nationality is associated with donor-earmarking passing from general to more specific subdimensions.

**Table 4.** IO head co-nationality and earmarking stringency (activity level).

	9 Stringency	10 Stringency	11 Stringency	12 Stringency
Co-nationality	0.088*** (0.004)	0.088*** (0.004)	0.074*** (0.005)	0.043*** (0.008)
Tenure (start)		−0.013*** (0.003)	−0.007* (0.003)	−0.007* (0.003)
Tenure (end)		0.005 ^+^ (0.003)	0.013*** (0.003)	0.014*** (0.003)
Size of earmarked contribution (log)			−0.015*** (0.001)	−0.014*** (0.001)
Number of donor-funded projects in IO (yearly, log)			0.004* (0.002)	0.002 (0.002)
Donor earmarked commitments to IO (yearly, log)			−0.009*** (0.001)	−0.008*** (0.001)
Donor total earmarked commitments (yearly, log, lagged)			−0.029*** (0.002)	−0.028*** (0.002)
Donor total multilateral commitments (yearly, log, lagged)			0.001 (0.003)	0.007* (0.003)
Donor total bilateral commitments (yearly, log, lagged)			0.071*** (0.005)	0.056*** (0.005)
Donor GDP per capita (log, lagged)			0.479*** (0.037)	0.500*** (0.037)
Donor democracy				0.983*** (0.039)
Donor ideal-point				−0.044*** (0.011)
Ideal-point distance				−0.008** (0.003)
Observations	110,420	110,420	74,594	74,189
R^2^	0.653	0.653	0.642	0.645
Fixed effects: IO	Yes	Yes	Yes	Yes
Fixed effects: Donor	Yes	Yes	Yes	Yes
Fixed effects: Recipient	Yes	Yes	Yes	Yes
Fixed effects: Year	Yes	Yes	Yes	Yes
Fixed effects: Sector	Yes	Yes	Yes	Yes

Clustered standard errors in parentheses.

+ *p* < 0.1, *p < 0.05, **p < 0.01, ***p < 0.001.

Table 5 presents models that include the interaction effect between the tenure and the nationality of executive heads. As H4–H9 expect, donor considerations over earmarked funding may be shaped by the leadership selection process in IOs. Donors’ efforts towards placing their candidates in executive office may involve promises of increased funding for specific programmes and geographic targets in a bid to curry favour with members and secure votes. Once their nationals enter executive office, donors may provide both larger earmarked contributions for individual IO activities and overall (yearly) volumes of earmarked funding to IOs (H4 and H5) as well as more stringently earmark contributions in a bid to keep on to their previous promises (H6). They might do so also when their nationals depart the executive office to align IO operations according to their promises to other members to secure their votes for the re-election of their national, or to bind future IO activities based on their preferences should a new non-national executive head enter office (H7–H9).

**Table 5.** Interaction between IO head co-nationality and tenure.

	13 Size	14 Volume	15 Stringency
Co-nationality	−0.456*** (0.048)	0.432*** (0.123)	0.050*** (0.009)
Tenure (start)	0.027 (0.021)	0.027 (0.048)	0.016*** (0.004)
Tenure (end)	−0.034 ^+^ (0.020)	0.032 (0.049)	0.001 (0.004)
Number of donor-funded projects in IO (yearly, log)	−0.973*** (0.010)	0.978*** (0.020)	0.002 (0.002)
Donor earmarked commitments to IO (yearly, log)	0.799*** (0.007)		−0.008*** (0.001)
Donor total earmarked commitments (yearly, log, lagged)	0.077*** (0.009)	0.130*** (0.024)	−0.030*** (0.002)
Donor total multilateral commitments (yearly, log, lagged)	−0.007 (0.016)	0.003 (0.048)	0.008** (0.003)
Donor total bilateral commitments (yearly, log, lagged)	−0.059* (0.026)	0.135* (0.062)	0.060*** (0.005)
Donor GDP per capita (log, lagged)	0.979*** (0.166)	1.629*** (0.368)	0.493*** (0.037)
Donor democracy	−0.929*** (0.249)	−0.761 (0.652)	0.985*** (0.040)
Donor ideal-point	0.123* (0.052)	−0.076 (0.161)	−0.038*** (0.011)
Ideal-point distance	−0.076*** (0.015)	−0.079* (0.035)	−0.007* (0.003)
Co-nationality × Tenure (start)	−0.190*** (0.045)	−0.125 (0.233)	−0.106*** (0.007)
Co-nationality × Tenure (end)	−0.030 (0.042)	−0.168 (0.231)	0.066*** (0.007)
Size of earmarked contribution (log)			−0.014*** (0.001)
Observations	74,351	7,326	74,189
R^2^	0.401	0.646	0.647
Fixed effects: IO	Yes	Yes	Yes
Fixed effects: Donor	Yes	Yes	Yes
Fixed effects: Recipient	Yes	No	Yes
Fixed effects: Year	Yes	Yes	Yes
Fixed effects: Sector	Yes	No	Yes

Clustered standard errors in parentheses.

+ *p* < 0.1, *p < 0.05, **p < 0.01, ***p < 0.001.

Model 13 includes the independent variable measuring the size of earmarked contributions provided by a donor for IO activities, together with all the control variables discussed earlier in Table 2 (and fixed effects) and the addition of two terms measuring: the interaction effect of co-nationality and the start of the tenure of executive heads and the interaction effect of co-nationality and the end of the tenure of executive heads. The coefficient of the interaction term for co-nationality and the start of the tenure of executive heads is negative and statistically significant (p < 0.001). The coefficient of the interaction term for co-nationality and the end of the tenure of executive heads is not statistically significant. The results from Model 13 indicate that, on average and relative to within-tenure years, donors decrease the size of their earmarked contributions to individual IO activities by approximately 17% (based on exponentiated coefficient) as their nationals enter executive office, and that the departure of their executive head has no significant effect on the size of their contributions. In other words, the results provide no support for H4 and H7.

Model 14 includes the independent variable measuring the yearly volume of donor-earmarked funding for IOs, together with all the control variables discussed earlier in Table 3 (and fixed effects) and the addition the interaction terms. Neither coefficients of the interaction terms are statistically significant, suggesting that the arrival or departure of their executive heads has no significant effect on the overall volume of donors’ contributions to IOs, in contrast to the expectations laid in H5 and H8.

Model 15 includes the independent variable measuring the stringency of earmarking in donor contributions to IO activities, together with all the control variables discussed in Table 4 (and fixed effects) as well as the two interaction terms of interest. The coefficient of the interaction term for co-nationality and the start of the tenure of executive heads is negative and statistically significant (*p* < 0.001), suggesting that, on average, donors decrease (rather than increase) the stringency of earmarking in their earmarked contributions as their nationals take the helm of the IO-executive office. This contrasts with the expectation of H6. However, the coefficient of the interaction term for co-nationality and the end of the tenure of executive heads is positive and statistically significant (*p* < 0.001), as expected in H9. In other words, the results suggest that, on average, donors increase the stringency of earmarking in their contributions for individual IO activities only as their nationals are about to leave the IO-executive office. These results, however, warrant caution in interpretation, as the interaction coefficients are very small and thus do not effectively imply any meaningful change in sub-dimensional earmarking.

We interpret the differential findings as implying that donors may prioritize one aspect of earmarking over another when strategically addressing the executive tenure of their nationals. It may be that providing larger earmarked contributions (and overall volumes of earmarked funding) may be seen as unwise in moments of uncertainty as the tenure of their nationals approaches its end, as doing so may not necessarily offer rewards. Donors instead seem to rely on earmarking their contributions more stringently, which would make these more effective at strategically inducing preference alignment. In more favourable times, such as immediately following the appointment of their nationals to IO-executive offices, donors seem to rely less on earmarked funding as a tool overall. In other words, while executive nationality is associated with covariation in donor engagement on all dimensions of earmarked funding, the moderating dynamics related to the stage of tenure of executive heads may be relevant only to moments of uncertainty, with donors potentially boosting their reliance on earmarked funding towards securing preference alignment beyond their nationals’ executive term.

Given that activities may be earmarked on more than one dimension (i.e. thematic, geographic and institutional), the earmarking stringency indicator on which the previous analysis relies represents an aggregated measure of various combinations of donor-earmarking. A more granular view on earmarking that distinguishes the specific dimensions of earmarking may aid in further understanding the effect of executive heads’ tenure on donor considerations for earmarking. Table 6 presents three additional models with stringency disaggregated along the dimensions of thematic, geographic and institutional earmarking.

**Table 6.** IO head co-nationality and earmarking stringency (disaggregated, activity level).

	16 Thematic	17 Geographic	18 Institutional
IO head co-nationality	0.041*** (0.008)	0.002 (0.003)	0.006 ^+^ (0.004)
Tenure (start)	0.017*** (0.004)	0.000 (0.001)	−0.002 (0.002)
Tenure (end)	0.004 (0.004)	−0.003 ^+^ (0.001)	−0.001 (0.002)
Size of earmarked contribution (log)	−0.002** (0.001)	0.000 (0.000)	−0.013*** (0.000)
Number of donor-funded projects in IO (yearly, log)	−0.003 (0.002)	0.003*** (0.001)	0.002* (0.001)
Donor total earmarked commitments to IO (yearly, log)	−0.006*** (0.001)	−0.001 (0.001)	−0.001 ^+^ (0.001)
Donor total earmarked commitments (yearly, log, lagged)	−0.032*** (0.002)	−0.002* (0.001)	0.004*** (0.001)
Donor multilateral commitments (yearly, log, lagged)	0.003 (0.003)	0.002* (0.001)	0.004** (0.001)
Donor bilateral commitments (yearly, log, lagged)	0.057*** (0.005)	−0.006** (0.002)	0.008*** (0.002)
Donor GDP per capita (log, lagged)	0.467*** (0.036)	0.004 (0.011)	0.021 (0.017)
Donor democracy	1.134*** (0.038)	0.049*** (0.014)	−0.196*** (0.019)
Donor ideal-point	−0.035*** (0.010)	0.012** (0.004)	−0.015*** (0.004)
Ideal-point distance	−0.013*** (0.003)	0.005*** (0.001)	0.002 (0.001)
Co-nationality × Tenure (start)	−0.107*** (0.007)	0.016*** (0.002)	−0.014*** (0.002)
Co-nationality × Tenure (end)	0.061*** (0.007)	0.014*** (0.002)	−0.009*** (0.002)
Observations	74,272	74,272	74,189
R^2^	0.266	0.933	0.245
Fixed effects: IO	Yes	Yes	Yes
Fixed effects: Donor	Yes	Yes	Yes
Fixed effects: Recipient	Yes	Yes	Yes
Fixed effects: Year	Yes	Yes	Yes
Fixed effects: Sector	Yes	Yes	Yes

Clustered standard errors in parentheses.

+ *p* < 0.1, *p < 0.05, **p < 0.01, ***p < 0.001.

Models 16, 17 and 18 include the independent variable measuring the stringency of earmarking disaggregated across the thematic, geographic and institutional dimensions in IO projects (respectively), together with all the control variables, fixed effects and the two interaction terms discussed earlier. The coefficients of the interaction term for co-nationality and the start of the tenure of executive heads are negative and statistically significant (*p* < 0.001) in Models 16 and 18, while the coefficient in Model 17 is positive and statistically significant (*p* < 0.001). In contrast, the coefficients of the interaction term for co-nationality and end of executive tenure are positive and statistically significant (*p* < 0.001) in Models 16 and 17, while the coefficient in Model 18 is negative and statistically significant (*p* < 0.001). While these results also warrant caution in interpretation as the coefficients are small and do not translate into meaningful change in sub-dimensional earmarking, they nevertheless suggest that the results on executive tenure in Model 15 (Table 5) may be driven, to some degree, by differential donor considerations towards specific dimensions of earmarking across individual IO activities.

The Supplemental Appendix presents additional analyses employed to probe the robustness of the findings. These generally support the validity of the main findings. Notably, the models in Table A7 estimate the interaction effect between donor ideology and executive head nationality on contribution size, overall (yearly) volume and stringency of earmarking. They suggest that the main results on size and stringency (at the activity level) may be driven by donor governments with an ideal-point closer to the US-led liberal order, while these governments appear to provide smaller overall volumes of earmarked funding (at the IO-donor-year level) when their nationals are in executive office. Supplemental Appendix Tables A9, A10 and A11 rely on samples limited to specific groups of countries (e.g. G7), suggesting similar implications to those of Supplemental Appendix Table A7 albeit with more mixed results. In general, whether donors engage differentially with earmarked funding when controlling IO executives seems to depend on whether they are broadly identifiable as Western countries, economically influential (e.g. being a member of G7/G12),^8^ or led by governments aligned with the US-led order (although the results are inconsistent when focusing only on the US itself). Importantly, these countries are also the largest contributors of earmarked funding within the multilateral system.

In sum, the findings suggest that the nationality and tenure of IO-executive heads is associated with variation in donor-earmarked funding. Congruence in nationality is associated negatively with the size of donor-earmarked contributions for IO activities, while it is positively associated with earmarking stringency and overall volume of earmarked funding provided. More fine-grained results suggest that donor considerations for earmarking may be driven not only by the nationality of executive heads, but also by their tenure.

## Conclusion

This article systematically examined the link between the control of IO-executive offices and variation in earmarked funding by member states. Both mechanisms are widely discussed in the IO literature as avenues of member state influence, but their relationship has not been systematically studied. The article expected donors to engage differentially with earmarked funding depending on whether their nationals held IO-executive offices. To empirically test its expectations regarding the covariation between IO-executive control and earmarked funding by donor states, the article relied on data on executive heads and earmarked funding in 49 IOs from 1990 to 2020. The findings suggest that the nationality of IO-executive heads is indeed associated with variation in earmarked funding. The findings suggest that executive representation is associated with, on average, smaller-sized donor contributions to individual IO activities and more stringent earmarking. Moreover, executive representation is associated with relatively larger overall (yearly) volumes of donor-earmarked funding.

In relation to the timing of executive tenure, the results offered mixed support for our expectations. First, donors appear to decrease the size of their earmarked contributions to individual IO activities when their nationals are entering office, while the end of their executive tenure is not associated with any change in contribution size. Executive tenure is also not associated with change in overall volume of donor-earmarked funding to IOs. Executive tenure appears to affect stringency with which donors earmark their individual contributions. Those results, however, warrant caution in interpretation as they do not effectively translate to meaningful change in sub-dimensional earmarking.

The findings carry several important implications. They are concerning given that most executive heads across multilaterals tend to be nationals of powerful and/or Western states (Novosad and Werker, 2019), which also make up the largest donor pool in terms of volume of voluntary funding that is earmarked, while the recipients of such funds are often poorer and less influential states. The afforded pathway for informal influence that such states may gain from having their nationals at the helms of IOs adds to their relatively privileged position (e.g. through voting power) within the multilateral system. This is even more problematic within the context of earmarked funding, although this study does not claim to offer causal evidence for donor influence.

Whether through their control of IO-executive offices or strategic reliance on earmarked funding, powerful states may be securing favourable outcomes at multilateral institutions while bypassing collective governance organs. In practice, this implies that multilateral institutions may be increasingly becoming implementing tools for already-influential states. This may be happening despite the recent rise in the authority of IOs (Zürn et al., 2021) and the increasing calls for reform aimed at providing non-Western states and less influential states with more voice in global governance.

Furthermore, earmarked funding has been shown to undermine the performance of IOs as compared to unearmarked core funding (Heinzel et al., 2023; Reinsberg and Siauwijaya, 2023). Previous studies have examined this effect by focusing only on the overall volume of earmarked funding provided by donors. The results here suggest that earmarking stringency may also be of importance. The variations in stringency observed here may suggest that it may be worth exploring other aspects and consequences of earmarked funding on IOs beyond volume and project performance. While unearmarked or even softly earmarked contributions may help expand IO resource pools without putting at risk the autonomy of IO bureaucracies in pursuing their tasks, the same may not be the case when it comes to strictly earmarked contributions. As the results here suggest, donors’ motivations towards earmarking their contributions may at least in part vary according to institutional dynamics. Future studies could benefit from accounting for such dynamics when examining the drivers of variation in earmarking stringency and its effects on IOs.

The article also makes several academic contributions to the study of IOs. Previous large-N studies on geographic representation and leadership selection in IO bureaucracies have mainly offered descriptive insights, explained states’ staffing practices by focusing on institutional design or examined the implications of states’ staffing practices in the wider bureaucracy of IOs rather than the executive level (Eckhard and Steinebach, 2021; Heinzel, 2022a, 2022b, 2022c; Novosad and Werker, 2019). Only a handful of studies have examined how the control of the executive level associates with variation in important IO outcomes, and they have mainly focused on specific cases such as the World Bank or the EU (Gehring and Schneider, 2018; Kaja and Werker, 2010). This article contributes to that literature by relying on a large sample of donors and IOs to offer new insights on how states’ control of IO-executive offices may potentially reflect on an important IO outcome, namely the allocation of earmarked resources for IO activities.

Related to the previous point, our findings speak to the literature on IO bureaucratic autonomy. Although it has been beyond the scope of our article to identify causal linkages, the findings suggest there to be at least a correlation between how states engage in earmarked funding and whether (and for how long) their nationals hold executive office in IOs. As such, we also interpret our findings as suggesting that IO-executive offices may be relevant enough to donor states to shape their resource allocation behaviour. They therefore encourage more research on whether states rely on alternative modalities of influence to keep IOs in line, such as through resource allocation. Extant studies have indeed highlighted the importance of resourcing dynamics to the autonomy of multilateral institutions (Ege and Bauer, 2017; Heldt and Schmidtke, 2017; Kilby, 2013). Despite the rise in authority of international bureaucracies in recent decades, IOs may not necessarily be immune to new modalities of state influence over their operations. We hope our findings will encourage more research exploring whether and how member states may be pursuing control over international bureaucracies through new funding modalities.

With that in mind, this article also acknowledges its limitations, which future research could address. First, the focus here has been on executive heads. However, previous studies have shown that powerful states also vie for control over other key positions in IO bureaucracies (Kleine, 2013). Future research could replicate the logic and findings of this article by accounting for a wider set of key offices within IOs. Second, revealing the mechanisms behind the causal influence of executive head nationality on donor states’ funding strategies has been beyond the scope of this paper. Future research could rely on qualitative approaches to shed light on how IO-executive head selection processes shape states’ considerations towards earmarking their contributions. Third, empirical patterns may vary considerably across the IOs in this sample, as may the pathways for influence. For example, in some IOs executive heads may hold more de-facto power, and some IOs have stricter fundraising and earmarking criteria than others. Some IO-specific features have been accounted for within the regression analyses of this study. Future studies could delve deeper into the variation in IO features to examine their potential effect on the interaction between member states’ informal influence and their earmarking arrangements with IOs. Fourth, due to data limitations, our analysis covers only activities that have been approved and/or implemented. This may imply a selection effect.

We also acknowledge alternative explanations. States’ choice between various modalities of overseas funding is expected to vary according to domestic concerns for aid effectiveness and political exigencies (Bayram and Graham, 2017; Eichenauer and Hug, 2018; Eichenauer and Reinsberg, 2017; Graham and Serdaru, 2020). Some of these dynamics, for example, political elections and turnover, may help explain the associated variation between donor-earmarking and executive control in IOs observed here, including those that contrasted our expectations. For example, when facing national elections and the risk of political turnover, incumbents in a donor state’s political system may engage more proactively with earmarked funding in IOs to future-proof the alignment of IO operations with their specific policy preferences. Newly elected governments may temporarily engage less with earmarked funding due to domestic policy priorities requiring the allocation of resources for particular policy initiatives back home. Future research could examine how such factors may moderate the effect of donors’ executive representation on their earmarking choices.

Finally, this article has focused on earmarked funding, which is often characterized by bilateral state-IO dynamics rather than collective decision-making. While this article has aimed at revealing a general phenomenon within a narrow empirical scope, future research may replicate the findings with different proxies for informal state influence and within different decision-making contexts. For example, future research could examine whether variation in states’ control of IO-executive offices is also associated with variation in unearmarked voluntary contributions. Future research could also examine systematically how executive head nationality – and other characteristics of executive heads, such as their tenure and prior experience in national offices – may be associated with policy- and decision-alignment with individual state interests within the context of IO collective decision-outcomes.

## Supplemental Material

sj-docx-1-ejt-10.1177_13540661251355045 – Supplemental material for Informal state influence in international organizations: examining the link between executive head nationality and earmarked funding Supplemental material, sj-docx-1-ejt-10.1177_13540661251355045 for Informal state influence in international organizations: examining the link between executive head nationality and earmarked funding by Giuseppe Zaccaria and Bernhard Reinsberg in European Journal of International Relations

## Notes

1. States sometimes even openly block the appointment of leadership candidates when they perceive their interests to be threatened. For example, the former UN High Commissioner for Human Rights, Mary Robinson, was critical of the Bush administration’s human rights record in the war on terror (Schulz, 2002). She later claimed she was blocked by that administration from continuing her position (Knowlton, 2002; Schechter, 2012).

2. In other words, we expect IO-executive heads to be incentivized towards capture and collusion while at the same time we expect IO rules, bureaucratic processes and other actors to discourage and even actively limit such outcomes (Stone, 2011). We do not claim to offer empirical evidence for our conjecture; instead, we rely on the insights of the studies reviewed here for this conjecture.

3. The monitoring requirements indicate the distrust between states and IOs and the need for addressing domestic pressures for transparency and budgetary control of multilateral funding.

4. Research shows that donors provide more multilateral aid to IOs that align with their preferences and that are more efficient (Schneider and Tobin, 2016). Our argument follows a similar logic but for earmarked funding volumes.

5. We are grateful to journal reviewers for encouraging us to expand our theorization and analysis of earmarked funding dynamics within the context of executive representation in IOs, specifically in regard to engaging with and developing expectations on executive tenure.

6. Breusch-Pagan tests reveal the presence of heteroskedasticity in the data. Thus, the regression models rely on robust standard errors.

7. Entries in that dataset end at the year mark of 2017 and do not contain information on all IOs and executive heads. The article expands these through researching and manually coding entries for missing IO-years.

8. The Group of 12 (G12) is composed of industrially advanced economies including Western countries (such as the US, the Netherlands, the UK) in addition to Japan. Together, these countries form the largest pool of contributors of earmarked funding within the multilateral system.
