# Supplementary material for: Informal state influence in international organizations: examining the link between executive head nationality and earmarked funding
Source: Eur J Int Relat. 2025 Jul 23;32(2):508–35. doi: 10.1177/13540661251355045 (PMC13167024; doi:10.1177/13540661251355045)
Supplement: sj-docx-1-ejt-10.1177_13540661251355045 – Supplemental material for Informal state influence in international organizations: examining the link between executive head nationality and earmarked funding [file sj-docx-1-ejt-10.1177_13540661251355045.docx]

Manuscript title:
Informal state influence in international organizations:
Examining the link between executive head nationality and earmarked funding

**Appendix**

**
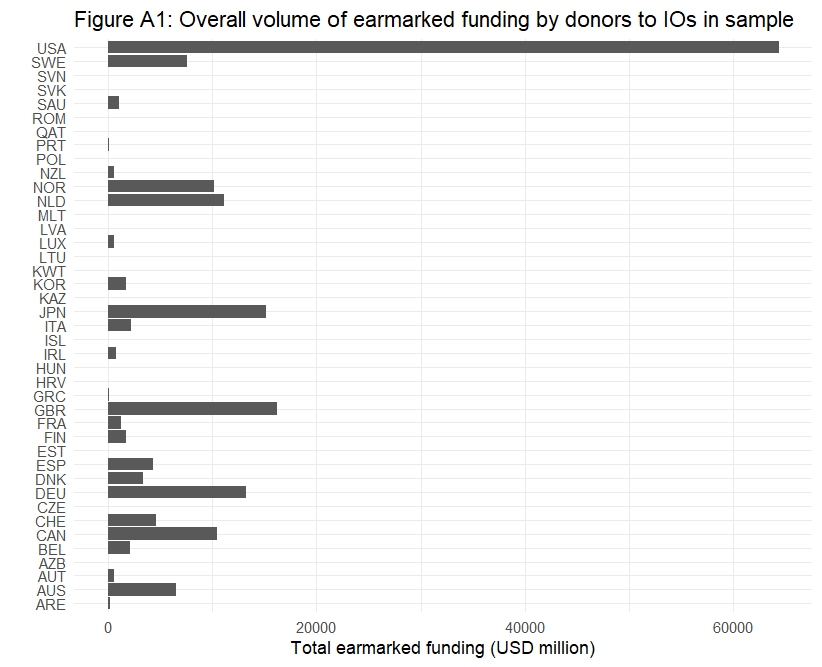
**

**
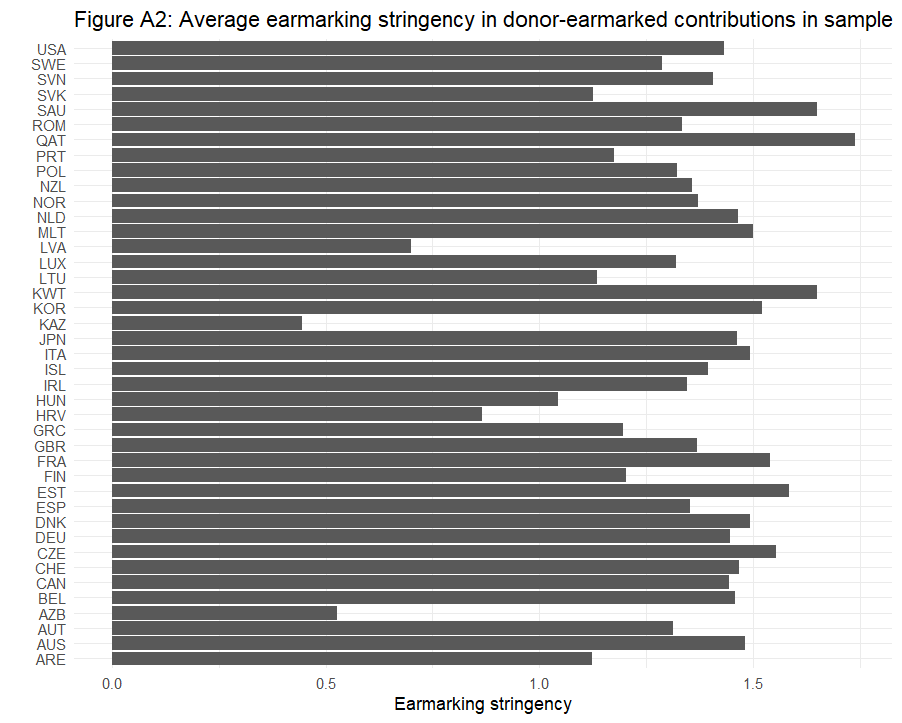
**

**
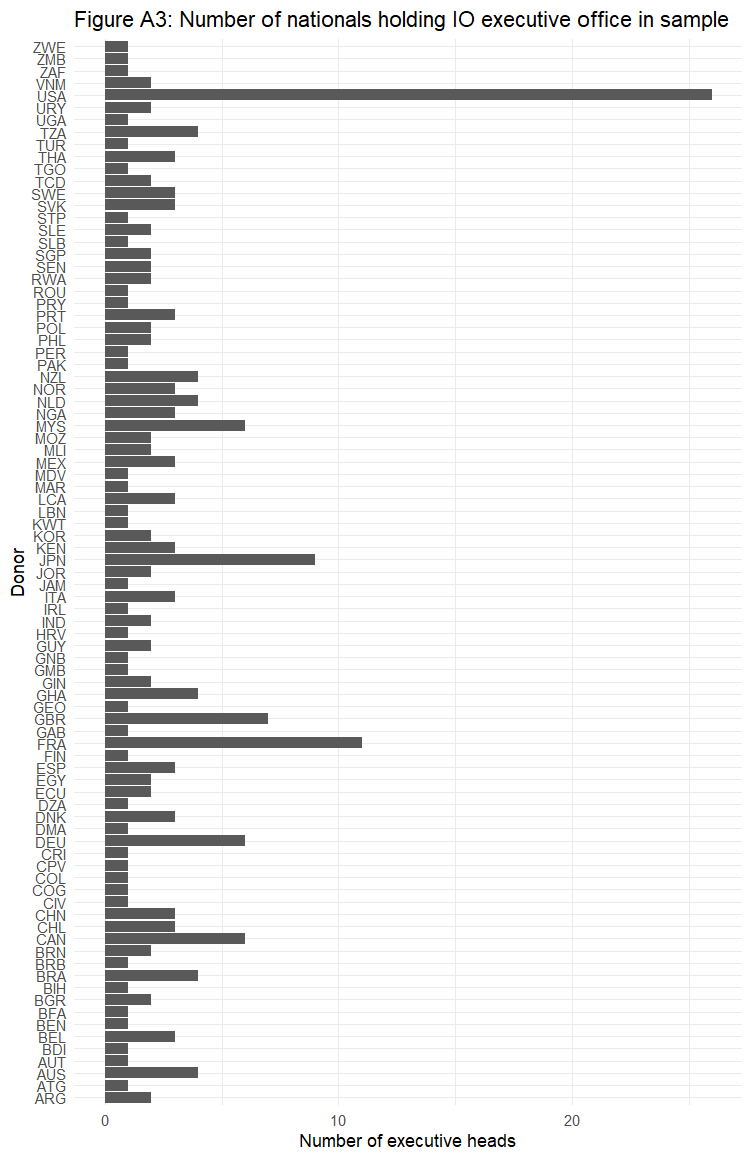
**

**
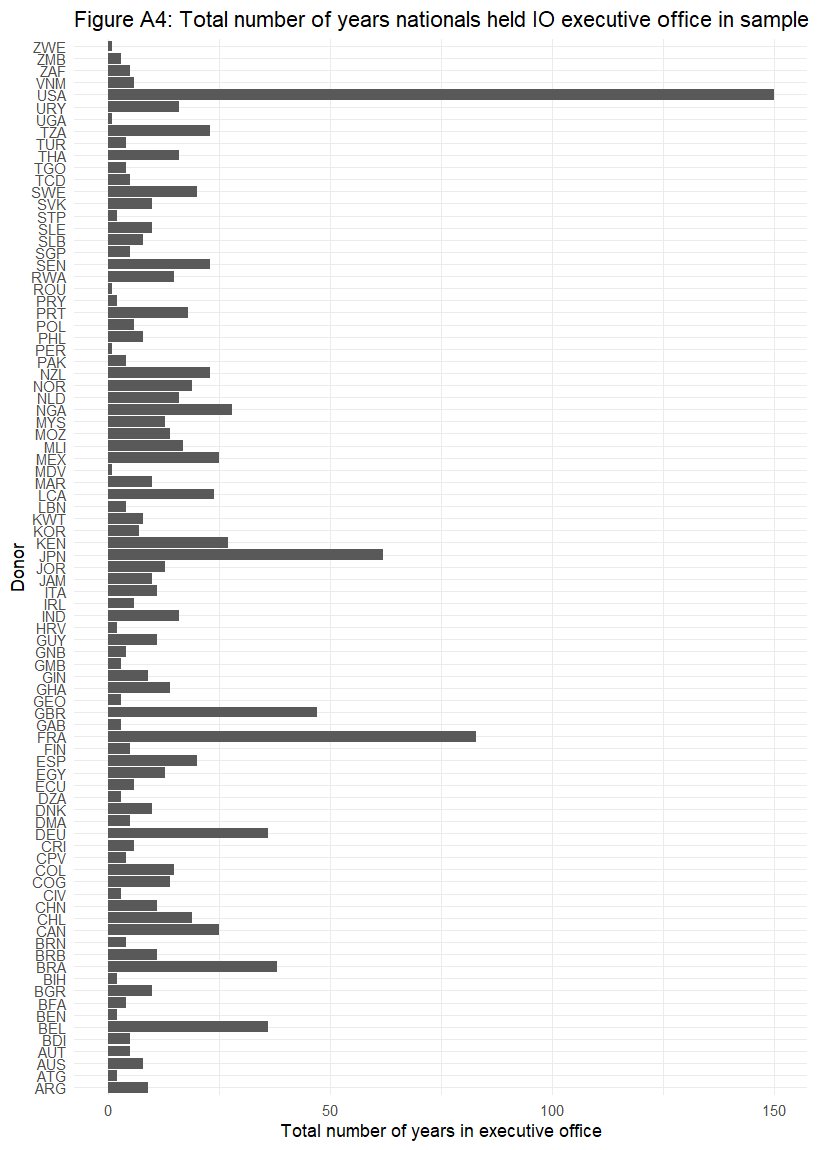
**

**
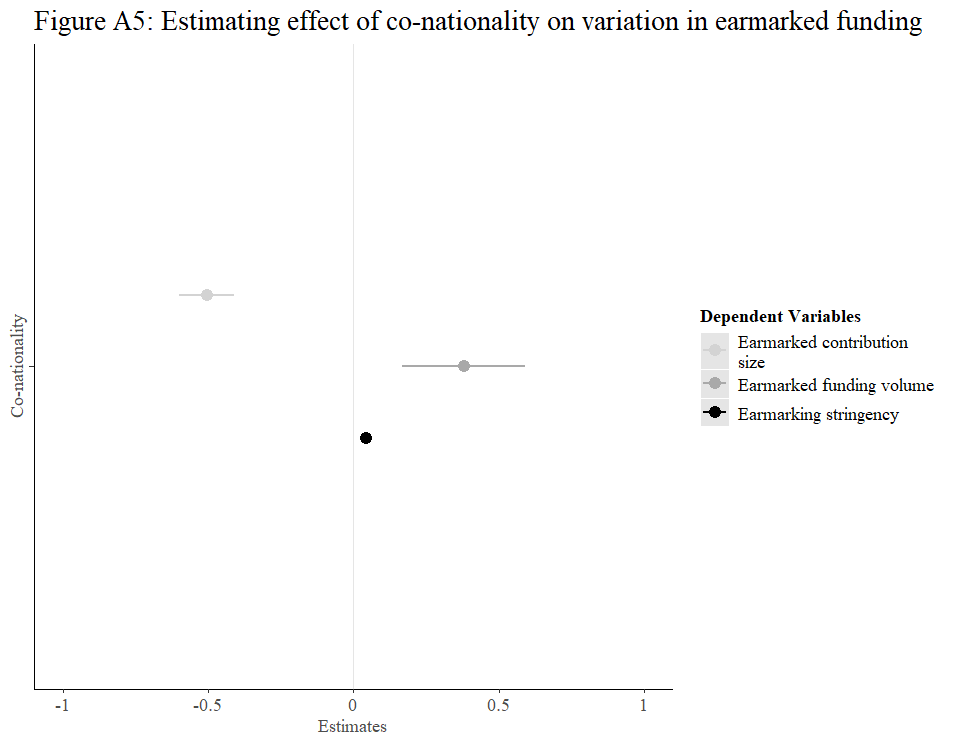
**

**
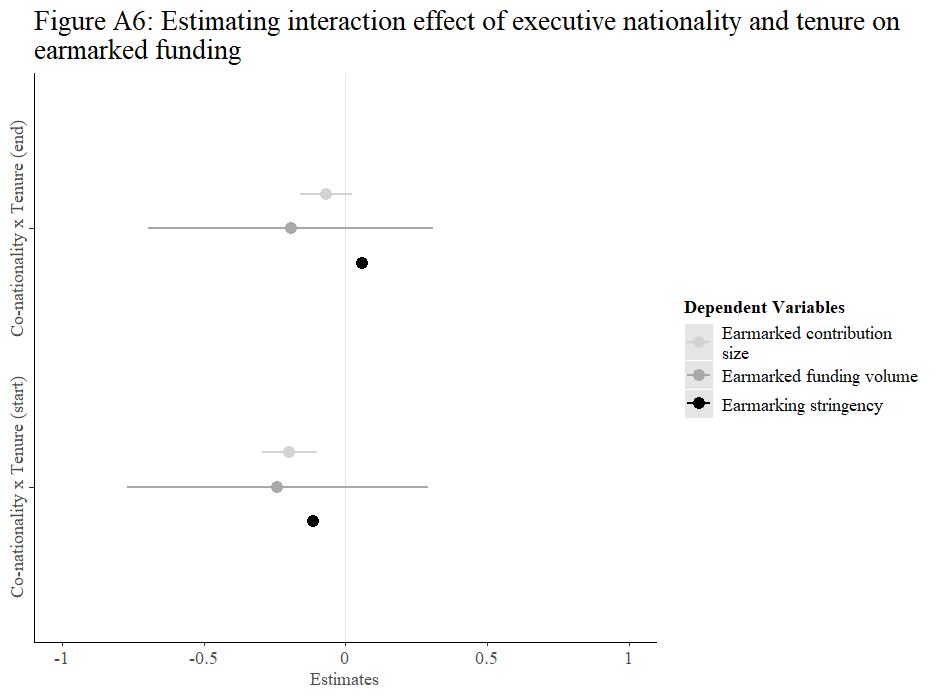
**

| **Table A1**. Alternative fixed effects specifications for model 4 (A1-A4) and model 13 (A5-A8). | | | | | | | | |
| --- | --- | --- | --- | --- | --- | --- | --- | --- |
|  | (A1) | (A2) | (A3) | (A4) | (A5) | (A6) | (A7) | (A8) |
| *IO head co-nationality* | -0.345*** | -0.639*** | -0.398*** | -0.490*** | -0.300*** | -0.597*** | -0.363*** | -0.453*** |
|  | (0.048) | (0.071) | (0.048) | (0.048) | (0.049) | (0.070) | (0.050) | (0.049) |
| *Tenure (start)* | -0.029 | -0.008 | 0.002 | -0.010 | 0.002 | 0.023 | 0.030 | 0.025 |
|  | (0.020) | (0.020) | (0.021) | (0.020) | (0.022) | (0.022) | (0.022) | (0.022) |
| *Tenure (end)* | -0.033+ | -0.040* | -0.017 | -0.021 | -0.030 | -0.039+ | -0.014 | -0.021 |
|  | (0.019) | (0.019) | (0.020) | (0.019) | (0.021) | (0.021) | (0.021) | (0.021) |
| *Number of donor-funded projects in IO (yearly, log)* | -0.925*** | -0.961*** | -0.926*** | -0.949*** | -0.926*** | -0.960*** | -0.925*** | -0.947*** |
|  | (0.011) | (0.013) | (0.010) | (0.010) | (0.011) | (0.013) | (0.010) | (0.010) |
| *Total volume of donor-earmarked funding in IO (yearly, log)* | 0.767*** | 0.761*** | 0.781*** | 0.781*** | 0.766*** | 0.760*** | 0.780*** | 0.780*** |
|  | (0.007) | (0.009) | (0.007) | (0.007) | (0.007) | (0.009) | (0.007) | (0.007) |
| *Donor earmarked commitments (yearly, log, lagged)* | 1.412 | 0.086*** | 0.061*** | 0.077*** | 1.565 | 0.085*** | 0.060*** | 0.076*** |
|  | (62720.480) | (0.009) | (0.009) | (0.009) | (62708.277) | (0.009) | (0.010) | (0.009) |
| *Donor multilateral commitments (yearly, log, lagged)* | -0.373 | -0.002 | -0.006 | -0.017 | -0.563 | -0.003 | -0.007 | -0.017 |
|  | (75161.024) | (0.016) | (0.017) | (0.016) | (75149.371) | (0.016) | (0.017) | (0.016) |
| *Ideal-point distance* | -0.055*** | -0.098*** | -0.052*** | -0.074*** | -0.055*** | -0.096*** | -0.052*** | -0.073*** |
|  | (0.015) | (0.021) | (0.015) | (0.015) | (0.015) | (0.021) | (0.015) | (0.015) |
| *Donor bilateral commitments (yearly, log, lagged)* |  | -0.014 | -0.045 | -0.084** |  | -0.012 | -0.043 | -0.081** |
|  |  | (0.027) | (0.028) | (0.027) |  | (0.027) | (0.028) | (0.027) |
| *Donor GDP per capita (log, lagged)* |  | 1.014*** | 0.868*** | 0.874*** |  | 1.013*** | 0.877*** | 0.876*** |
|  |  | (0.174) | (0.178) | (0.175) |  | (0.174) | (0.178) | (0.175) |
| Donor democracy |  | -0.409 | -1.188*** | -1.108*** |  | -0.498+ | -1.267*** | -1.195*** |
|  |  | (0.260) | (0.261) | (0.254) |  | (0.263) | (0.264) | (0.256) |
| *Donor ideal-point* |  | 0.166** | 0.206*** | 0.122* |  | 0.165** | 0.206*** | 0.121* |
|  |  | (0.054) | (0.057) | (0.055) |  | (0.054) | (0.057) | (0.055) |
| *Co-nationality x tenure (start)* |  |  |  |  | -0.194*** | -0.142** | -0.149** | -0.167*** |
|  |  |  |  |  | (0.056) | (0.045) | (0.047) | (0.044) |
| *Co-nationality x tenure (end)* |  |  |  |  | -0.032 | -0.006 | -0.021 | -0.008 |
|  |  |  |  |  | (0.051) | (0.042) | (0.045) | (0.042) |
| Observations | 74351 | 74351 | 74351 | 74351 | 74351 | 74351 | 74351 | 74351 |
| R^2^ | 0.420 | 0.414 | 0.449 | 0.455 | 0.421 | 0.414 | 0.449 | 0.455 |
| Fixed effects: Donor | No | No | Yes | Yes | No | No | Yes | Yes |
| Fixed effects: IO | Yes | No | Yes | Yes | Yes | No | Yes | Yes |
| Fixed effects: Year | No | Yes | No | Yes | No | Yes | No | Yes |
| Fixed effects: Recipient-Sector | No | No | No | Yes | No | No | No | Yes |
| Fixed effects: Recipient-Year | No | No | Yes | No | No | No | Yes | No |
| Fixed effects: Sector | Yes | Yes | Yes | No | Yes | Yes | Yes | No |
| Fixed effects: Recipient | Yes | Yes | No | No | Yes | Yes | No | No |
| Fixed effects: Donor-Year | Yes | No | No | No | Yes | No | No | No |
| Fixed effects: IO-Donor | No | Yes | No | No | No | Yes | No | No |
| *Clustered standard errors in parentheses; + p < 0.1, * p < 0.05, ** p < 0.01, *** p < 0.001* | | | | | | | | |

| **Table A2**. Alternative fixed effects specifications for model 8 (A9-A10) and model 14 (A11-A12). | | | | |
| --- | --- | --- | --- | --- |
|  | (A9) | (A10) | (A11) | (A12) |
| *Executive co-nationality* | 0.421*** | 0.277* | 0.461*** | 0.350* |
|  | (0.109) | (0.126) | (0.127) | (0.143) |
| *Tenure (start)* | -0.007 |  | -0.006 |  |
|  | (0.047) |  | (0.048) |  |
| *Tenure (end)* | 0.022 |  | 0.028 |  |
|  | (0.048) |  | (0.049) |  |
| *Number of donor-funded projects in IO (yearly, log)* | 1.018*** | 0.978*** | 1.018*** | 0.978*** |
|  | (0.021) | (0.021) | (0.021) | (0.021) |
| *Ideal-point distance* | -0.079* | -0.114* | -0.079* | -0.115* |
|  | (0.035) | (0.051) | (0.035) | (0.051) |
| *Donor earmarked commitments (yearly, log, lagged)* |  | 0.146*** |  | 0.146*** |
|  |  | (0.025) |  | (0.025) |
| *Donor multilateral commitments (yearly, log, lagged)* |  | -0.001 |  | -0.001 |
|  |  | (0.047) |  | (0.047) |
| *Donor bilateral commitments (yearly, log, lagged)* |  | 0.131* |  | 0.132* |
|  |  | (0.064) |  | (0.064) |
| *Donor GDP per capita (log, lagged)* |  | 1.418*** |  | 1.418*** |
|  |  | (0.386) |  | (0.386) |
| *Donor democracy* |  | -0.834 |  | -0.852 |
|  |  | (0.699) |  | (0.701) |
| *Donor ideal-point* |  | 0.041 |  | 0.038 |
|  |  | (0.166) |  | (0.166) |
| *Co-nationality x tenure (start)* |  |  | -0.022 | -0.208 |
|  |  |  | (0.253) | (0.250) |
| *Co-nationality x tenure (end)* |  |  | -0.191 | -0.219 |
|  |  |  | (0.236) | (0.255) |
| Observations | 7326 | 7326 | 7326 | 7326 |
| R^2^ | 0.685 | 0.703 | 0.685 | 0.703 |
| Fixed effects: Donor | No | Yes | No | Yes |
| Fixed effects: IO-Year | No | Yes | No | Yes |
| Fixed effects: IO | Yes | No | Yes | No |
| Fixed effects: Donor-Year | Yes | No | Yes | No |
| *Clustered standard errors in parentheses; + p < 0.1, * p < 0.05, ** p < 0.01, *** p < 0.001* | | | | |

| **Table A3.** Alternative fixed effects specifications for model 12 (A13-A16) and model 15 (A17-A20). | | | | | | | | |
| --- | --- | --- | --- | --- | --- | --- | --- | --- |
|  | (A13) | (A14) | (A15) | (A16) | (A17) | (A18) | (A19) | (A20) |
| *Executive co-nationality* | 0.023** | 0.024* | 0.039*** | 0.044*** | 0.021* | 0.038*** | 0.044*** | 0.047*** |
|  | (0.008) | (0.011) | (0.008) | (0.009) | (0.009) | (0.012) | (0.009) | (0.009) |
| *Tenure (start)* | 0.015*** | -0.012*** | -0.003 | -0.010** | 0.022*** | 0.013*** | 0.015*** | 0.012** |
|  | (0.003) | (0.003) | (0.004) | (0.003) | (0.004) | (0.004) | (0.004) | (0.004) |
| *Tenure (end)* | 0.015*** | 0.012*** | 0.005 | 0.013*** | 0.008* | -0.001 | -0.004 | -0.001 |
|  | (0.003) | (0.003) | (0.004) | (0.004) | (0.004) | (0.004) | (0.004) | (0.004) |
| *Size of donor-earmarked contribution (log)* | -0.012*** | -0.013*** | -0.015*** | -0.015*** | -0.012*** | -0.013*** | -0.015*** | -0.015*** |
|  | (0.001) | (0.001) | (0.001) | (0.001) | (0.001) | (0.001) | (0.001) | (0.001) |
| *Number of donor-funded projects in IO (yearly, log)* | 0.002 | -0.018*** | 0.002 | 0.001 | 0.002 | -0.017*** | 0.002 | 0.001 |
|  | (0.002) | (0.002) | (0.002) | (0.002) | (0.002) | (0.003) | (0.002) | (0.002) |
| *Total volume of donor-earmarked funding in IO (yearly, log)* | -0.003+ | -0.013*** | -0.006*** | -0.006*** | -0.003+ | -0.014*** | -0.006*** | -0.006*** |
|  | (0.001) | (0.002) | (0.001) | (0.001) | (0.001) | (0.002) | (0.001) | (0.001) |
| *Donor earmarked commitments (yearly, log, lagged)* | 1.457 | -0.020*** | -0.028*** | -0.028*** | 1.460 | -0.023*** | -0.030*** | -0.030*** |
|  | (10562.043) | (0.002) | (0.002) | (0.002) | (10564.101) | (0.002) | (0.002) | (0.002) |
| *Donor multilateral commitments (yearly, log, lagged)* | -0.703 | 0.008** | -0.001 | 0.005+ | -0.707 | 0.008** | -0.001 | 0.006* |
|  | (10804.145) | (0.003) | (0.003) | (0.003) | (10814.236) | (0.003) | (0.003) | (0.003) |
| *Ideal-point distance* | -0.006* | -0.007+ | -0.007* | -0.008** | -0.006* | -0.005 | -0.007* | -0.008** |
|  | (0.003) | (0.004) | (0.003) | (0.003) | (0.003) | (0.004) | (0.003) | (0.003) |
| *Donor bilateral commitments (yearly, log, lagged)* |  | 0.059*** | 0.074*** | 0.054*** |  | 0.062*** | 0.078*** | 0.058*** |
|  |  | (0.005) | (0.006) | (0.006) |  | (0.005) | (0.006) | (0.006) |
| *Donor GDP per capita (log, lagged)* |  | 0.465*** | 0.538*** | 0.431*** |  | 0.456*** | 0.535*** | 0.424*** |
|  |  | (0.038) | (0.039) | (0.038) |  | (0.038) | (0.039) | (0.038) |
| *Donor democracy* |  | 0.972*** | 0.996*** | 1.014*** |  | 0.956*** | 0.992*** | 1.019*** |
|  |  | (0.040) | (0.041) | (0.041) |  | (0.041) | (0.042) | (0.042) |
| *Donor ideal-point* |  | -0.036** | -0.021+ | -0.012 |  | -0.031** | -0.016 | -0.005 |
|  |  | (0.011) | (0.012) | (0.011) |  | (0.011) | (0.012) | (0.011) |
| *Co-nationality x tenure (start)* |  |  |  |  | -0.042*** | -0.114*** | -0.096*** | -0.100*** |
|  |  |  |  |  | (0.008) | (0.007) | (0.007) | (0.007) |
| *Co-nationality x tenure (end)* |  |  |  |  | 0.040*** | 0.062*** | 0.055*** | 0.070*** |
|  |  |  |  |  | (0.007) | (0.007) | (0.007) | (0.007) |
| Observations | 74189 | 74189 | 74189 | 74189 | 74189 | 74189 | 74189 | 74189 |
| R^2^ | 0.703 | 0.667 | 0.685 | 0.672 | 0.704 | 0.669 | 0.686 | 0.674 |
| Fixed effects: Donor | No | No | Yes | Yes | No | No | Yes | Yes |
| Fixed effects: IO | Yes | No | Yes | Yes | Yes | No | Yes | Yes |
| Fixed effects: year | No | Yes | No | Yes | No | Yes | No | Yes |
| Fixed effects: Recipient-Sector | No | No | No | Yes | No | No | No | Yes |
| Fixed effects: Recipient-Year | No | No | Yes | No | No | No | Yes | No |
| Fixed effects: Sector | Yes | Yes | Yes | No | Yes | Yes | Yes | No |
| Fixed effects: Recipient | Yes | Yes | No | No | Yes | Yes | No | No |
| Fixed effects: Donor-Year | Yes | No | No | No | Yes | No | No | No |
| Fixed effects: IO-Donor | No | Yes | No | No | No | Yes | No | No |
| *Clustered standard errors in parentheses; + p < 0.1, * p < 0.05, ** p < 0.01, *** p < 0.001* | | | | | | | | |

| **Table A4.** Alternative fixed effects specifications for model 16. | | | | |
| --- | --- | --- | --- | --- |
|  | (A21) | (A22) | (A23) | (A24) |
| *Executive co-nationality* | 0.014+ | 0.024* | 0.034*** | 0.036*** |
|  | (0.008) | (0.011) | (0.009) | (0.009) |
| *Tenure (start)* | 0.024*** | 0.018*** | 0.017*** | 0.012** |
|  | (0.004) | (0.004) | (0.004) | (0.004) |
| *Tenure (end)* | 0.013*** | 0.003 | 0.000 | 0.003 |
|  | (0.004) | (0.004) | (0.004) | (0.004) |
| *Size of earmarked contribution (log)* | 0.001 | -0.001 | -0.002** | -0.003*** |
|  | (0.001) | (0.001) | (0.001) | (0.001) |
| *Number of donor-funded projects in IO (yearly, log)* | -0.003 | -0.017*** | -0.002 | -0.003 |
|  | (0.002) | (0.002) | (0.002) | (0.002) |
| *Total volume of donor-earmarked funding in IO (yearly, log)* | 0.001 | -0.012*** | -0.005** | -0.005*** |
|  | (0.001) | (0.002) | (0.001) | (0.001) |
| *Donor earmarked commitments (yearly, log, lagged)* | 0.154 | -0.025*** | -0.031*** | -0.032*** |
|  | (10538.776) | (0.002) | (0.002) | (0.002) |
| *Donor multilateral commitments (yearly, log, lagged)* | -0.298 | 0.002 | -0.007* | 0.001 |
|  | (10645.005) | (0.003) | (0.003) | (0.003) |
| *Ideal-point distance* | -0.012*** | -0.014*** | -0.014*** | -0.014*** |
|  | (0.003) | (0.004) | (0.003) | (0.003) |
| *Co-nationality x tenure (end)* | 0.028*** | 0.057*** | 0.047*** | 0.065*** |
|  | (0.007) | (0.007) | (0.007) | (0.007) |
| *Co-nationality x tenure (start)* | -0.047*** | -0.119*** | -0.103*** | -0.101*** |
|  | (0.008) | (0.007) | (0.007) | (0.007) |
| *Donor bilateral commitments (yearly, log, lagged)* |  | 0.057*** | 0.076*** | 0.058*** |
|  |  | (0.005) | (0.006) | (0.005) |
| *Donor GDP per capita (log, lagged)* |  | 0.398*** | 0.505*** | 0.406*** |
|  |  | (0.037) | (0.038) | (0.038) |
| *Donor democracy* |  | 1.048*** | 1.160*** | 1.158*** |
|  |  | (0.039) | (0.041) | (0.040) |
| *Donor ideal-point* |  | -0.031** | -0.006 | -0.011 |
|  |  | (0.010) | (0.011) | (0.011) |
| Observations | 74272 | 74272 | 74272 | 74272 |
| R^2^ | 0.388 | 0.318 | 0.345 | 0.327 |
| Fixed effects: IO | Yes | No | Yes | Yes |
| Fixed effects: Year | No | Yes | No | Yes |
| Fixed effects: Donor | No | No | Yes | Yes |
| Fixed effects: Sector | Yes | Yes | Yes | No |
| Fixed effects: Donor-Year | Yes | No | No | No |
| Fixed effects: Recipient | Yes | Yes | No | No |
| Fixed effects: IO-Donor | No | Yes | No | No |
| Fixed effects: Recipient-Year | No | No | Yes | No |
| Fixed effects: Recipient-Sector | No | No | No | Yes |
| *Clustered standard errors in parentheses; + p < 0.1, * p < 0.05, ** p < 0.01, *** p < 0.001* | | | | |

| **Table A5.** Alternative fixed effects specifications for model 17. | | | | |
| --- | --- | --- | --- | --- |
|  | (A25) | (A26) | (A27) | (A28) |
| *Executive co-nationality* | 0.004 | -0.000 | 0.005 | 0.005 |
|  | (0.003) | (0.005) | (0.003) | (0.003) |
| *Tenure (start)* | 0.001 | 0.000 | 0.000 | 0.001 |
|  | (0.001) | (0.001) | (0.001) | (0.001) |
| *Tenure (end)* | -0.003* | -0.002+ | -0.004* | -0.002 |
|  | (0.001) | (0.001) | (0.001) | (0.001) |
| *Size of earmarked contribution (log)* | 0.000 | 0.000 | -0.000 | 0.000 |
|  | (0.000) | (0.000) | (0.000) | (0.000) |
| *Number of donor-funded projects in IO (yearly, log)* | 0.002+ | 0.001 | 0.002** | 0.003** |
|  | (0.001) | (0.001) | (0.001) | (0.001) |
| *Total volume of donor-earmarked funding in IO (yearly, log)* | -0.001 | -0.000 | -0.000 | -0.000 |
|  | (0.001) | (0.001) | (0.001) | (0.001) |
| *Donor earmarked commitments (yearly, log, lagged)* | 1.609 | -0.002+ | -0.002** | -0.002+ |
|  | (3990.124) | (0.001) | (0.001) | (0.001) |
| *Donor multilateral commitments (yearly, log, lagged)* | -0.860 | 0.002* | 0.002* | 0.002* |
|  | (4467.902) | (0.001) | (0.001) | (0.001) |
| *Ideal-point distance* | 0.004*** | 0.004** | 0.005*** | 0.005*** |
|  | (0.001) | (0.001) | (0.001) | (0.001) |
| *Co-nationality x tenure (end)* | 0.010*** | 0.012*** | 0.013*** | 0.013*** |
|  | (0.002) | (0.002) | (0.002) | (0.002) |
| *Co-nationality x tenure (start)* | 0.014*** | 0.014*** | 0.016*** | 0.015*** |
|  | (0.003) | (0.002) | (0.002) | (0.002) |
| *Donor bilateral commitments (yearly, log, lagged)* |  | -0.003+ | -0.006** | -0.006** |
|  |  | (0.002) | (0.002) | (0.002) |
| *Donor GDP per capita (log, lagged)* |  | 0.009 | 0.013 | -0.006 |
|  |  | (0.011) | (0.012) | (0.012) |
| *Donor democracy* |  | 0.058*** | 0.034* | 0.051*** |
|  |  | (0.014) | (0.015) | (0.015) |
| *Donor ideal-point* |  | 0.012** | 0.010* | 0.014*** |
|  |  | (0.004) | (0.005) | (0.004) |
| Observations | 74272 | 74272 | 74272 | 74272 |
| R^2^ | 0.935 | 0.938 | 0.938 | 0.936 |
| Fixed effects: IO | Yes | No | Yes | Yes |
| Fixed effects: Year | No | Yes | No | Yes |
| Fixed effects: Donor | No | No | Yes | Yes |
| Fixed effects: Sector | Yes | Yes | Yes | No |
| Fixed effects: Donor-Year | Yes | No | No | No |
| Fixed effects: Recipient | Yes | Yes | No | No |
| Fixed effects: IO-Donor | No | Yes | No | No |
| Fixed effects: Recipient-Year | No | No | Yes | No |
| Fixed effects: Recipient-Sector | No | No | No | Yes |
| *Clustered standard errors in parentheses; + p < 0.1, * p < 0.05, ** p < 0.01, *** p < 0.001* | | | | |

| **Table A6.** Alternative fixed effects specifications for model 18. | | | | |
| --- | --- | --- | --- | --- |
|  | (A29) | (A30) | (A31) | (A32) |
| *Executive co-nationality* | 0.003 | 0.014** | 0.006 | 0.007+ |
|  | (0.004) | (0.005) | (0.004) | (0.004) |
| *Tenure (start)* | -0.003 | -0.005** | -0.002 | -0.001 |
|  | (0.002) | (0.002) | (0.002) | (0.002) |
| *Tenure (end)* | -0.002 | -0.001 | -0.001 | -0.002 |
|  | (0.002) | (0.002) | (0.002) | (0.002) |
| *Size of earmarked contribution (log)* | -0.013*** | -0.013*** | -0.013*** | -0.012*** |
|  | (0.000) | (0.000) | (0.000) | (0.000) |
| *Number of donor-funded projects in IO (yearly, log)* | 0.003** | -0.001 | 0.002* | 0.001 |
|  | (0.001) | (0.001) | (0.001) | (0.001) |
| *Total volume of donor-earmarked funding in IO (yearly, log)* | -0.002** | -0.002+ | -0.001+ | -0.001 |
|  | (0.001) | (0.001) | (0.001) | (0.001) |
| *Donor earmarked commitments (yearly, log, lagged)* | -0.622 | 0.004*** | 0.003*** | 0.003*** |
|  | (4647.257) | (0.001) | (0.001) | (0.001) |
| *Donor multilateral commitments (yearly, log, lagged)* | 0.864 | 0.005*** | 0.004** | 0.004* |
|  | (4731.554) | (0.001) | (0.002) | (0.001) |
| *Ideal-point distance* | 0.001 | 0.005** | 0.002+ | 0.001 |
|  | (0.001) | (0.002) | (0.001) | (0.001) |
| *Co-nationality x tenure (end)* | 0.000 | -0.008*** | -0.006* | -0.008*** |
|  | (0.002) | (0.002) | (0.002) | (0.002) |
| *Co-nationality x tenure (start)* | -0.008** | -0.009*** | -0.010*** | -0.013*** |
|  | (0.003) | (0.002) | (0.003) | (0.002) |
| *Donor bilateral commitments (yearly, log, lagged)* |  | 0.009*** | 0.007** | 0.006** |
|  |  | (0.002) | (0.002) | (0.002) |
| *Donor GDP per capita (log, lagged)* |  | 0.048** | 0.017 | 0.024 |
|  |  | (0.017) | (0.018) | (0.017) |
| *Donor democracy* |  | -0.148*** | -0.202*** | -0.190*** |
|  |  | (0.018) | (0.020) | (0.019) |
| *Donor ideal-point* |  | -0.013** | -0.020*** | -0.010* |
|  |  | (0.004) | (0.005) | (0.004) |
| Observations | 74189 | 74189 | 74189 | 74189 |
| R^2^ | 0.286 | 0.307 | 0.300 | 0.316 |
| Fixed effects: IO | Yes | No | Yes | Yes |
| Fixed effects: Year | No | Yes | No | Yes |
| Fixed effects: Donor | No | No | Yes | Yes |
| Fixed effects: Sector | Yes | Yes | Yes | No |
| Fixed effects: Donor-Year | Yes | No | No | No |
| Fixed effects: Recipient | Yes | Yes | No | No |
| Fixed effects: IO-Donor | No | Yes | No | No |
| Fixed effects: Recipient-Year | No | No | Yes | No |
| Fixed effects: Recipient-Sector | No | No | No | Yes |
| *Clustered standard errors in parentheses; + p < 0.1, * p < 0.05, ** p < 0.01, *** p < 0.001* | | | | |

| **Table A7.** Interaction between co-nationality and donor ideal-point. | | | | | | |
| --- | --- | --- | --- | --- | --- | --- |
|  | (A33 size) | (A34 vol) | (A35 str) | (A36 thm) | (A37 geo) | (A38 inst) |
| *Executive co-nationality* | 0.094 | 0.817*** | -0.155*** | -0.121*** | -0.021** | -0.012 |
|  | (0.123) | (0.223) | (0.021) | (0.021) | (0.008) | (0.009) |
| *Tenure (start)* | -0.021 | 0.022 | -0.004 | -0.004 | 0.004*** | -0.004** |
|  | (0.019) | (0.048) | (0.003) | (0.003) | (0.001) | (0.002) |
| *Tenure (end)* | -0.040* | 0.028 | 0.014*** | 0.017*** | 0.000 | -0.003+ |
|  | (0.019) | (0.048) | (0.003) | (0.003) | (0.001) | (0.002) |
| *Number of donor-funded projects in IO (yearly, log)* | -0.967*** | 0.981*** | -0.001 | -0.005** | 0.003*** | 0.002* |
|  | (0.010) | (0.020) | (0.002) | (0.002) | (0.001) | (0.001) |
| *Total volume of donor-earmarked funding in IO (yearly, log)* | 0.795*** |  | -0.006*** | -0.004** | -0.001 | -0.001 |
|  | (0.007) |  | (0.001) | (0.001) | (0.001) | (0.001) |
| *Donor earmarked commitments (yearly, log, lagged)* | 0.077*** | 0.129*** | -0.027*** | -0.029*** | -0.001+ | 0.004*** |
|  | (0.009) | (0.024) | (0.002) | (0.002) | (0.001) | (0.001) |
| *Donor multilateral commitments (yearly, log, lagged)* | -0.006 | 0.004 | 0.007* | 0.002 | 0.002+ | 0.004** |
|  | (0.016) | (0.048) | (0.003) | (0.003) | (0.001) | (0.001) |
| *Donor bilateral commitments (yearly, log, lagged)* | -0.057* | 0.133* | 0.055*** | 0.053*** | -0.006*** | 0.008*** |
|  | (0.026) | (0.062) | (0.005) | (0.005) | (0.002) | (0.002) |
| *Donor GDP per capita (log, lagged)* | 1.086*** | 1.658*** | 0.464*** | 0.446*** | -0.000 | 0.018 |
|  | (0.167) | (0.369) | (0.037) | (0.036) | (0.011) | (0.017) |
| *Donor democracy* | -0.794** | -0.737 | 0.975*** | 1.130*** | 0.030* | -0.184*** |
|  | (0.246) | (0.651) | (0.039) | (0.038) | (0.014) | (0.018) |
| *Donor ideal-point* | 0.169** | -0.057 | -0.058*** | -0.052*** | 0.008* | -0.015*** |
|  | (0.053) | (0.161) | (0.011) | (0.010) | (0.004) | (0.004) |
| *Ideal-point distance* | -0.117*** | -0.105** | 0.005+ | -0.004 | 0.007*** | 0.002+ |
|  | (0.016) | (0.037) | (0.003) | (0.003) | (0.001) | (0.001) |
| *Co-nationality x donor ideal-point* | -0.300*** | -0.299* | 0.099*** | 0.077*** | 0.015*** | 0.007+ |
|  | (0.053) | (0.135) | (0.009) | (0.009) | (0.003) | (0.004) |
| *Size of earmarked contribution (log)* |  |  | -0.014*** | -0.001* | 0.000 | -0.013*** |
|  |  |  | (0.001) | (0.001) | (0.000) | (0.000) |
| Observations | 74351 | 7326 | 74189 | 74272 | 74272 | 74189 |
| R^2^ | 0.401 | 0.646 | 0.646 | 0.263 | 0.933 | 0.245 |
| Fixed effects: IO | Yes | Yes | Yes | Yes | Yes | Yes |
| Fixed effects: Donor | Yes | Yes | Yes | Yes | Yes | Yes |
| Fixed effects: Year | Yes | Yes | Yes | Yes | Yes | Yes |
| Fixed effects: Sector | Yes | No | Yes | Yes | Yes | Yes |
| Fixed effects: Recipient | Yes | No | Yes | Yes | Yes | Yes |
| *Clustered standard errors in parentheses; + p < 0.1, * p < 0.05, ** p < 0.01, *** p < 0.001* | | | | | | |

| **Table A8.** Alternative sampling for models 4, 8, and 12. | | | | | | |
| --- | --- | --- | --- | --- | --- | --- |
|  | (Size: conationality) | (Size: no conationality) | (Volume: conationality) | (Volume: no conationality) | (Stringency: conationality) | (Stringency: no conationality) |
| *Tenure (start)* | -0.090 | 0.008 | -0.030 | 0.023 | -0.022* | 0.012** |
|  | (0.056) | (0.022) | (0.164) | (0.049) | (0.010) | (0.004) |
| *Tenure (end)* | 0.005 | -0.049* | -0.170 | 0.037 | 0.045*** | 0.008* |
|  | (0.055) | (0.021) | (0.187) | (0.049) | (0.008) | (0.004) |
| *Number of donor-funded projects in IO (yearly, log)* | -1.041*** | -0.920*** | 0.947*** | 0.977*** | 0.060*** | -0.011*** |
|  | (0.051) | (0.010) | (0.131) | (0.021) | (0.007) | (0.002) |
| *Total volume of donor-earmarked funding in IO (yearly, log)* | 0.608*** | 0.789*** |  |  | -0.037*** | 0.002 |
|  | (0.062) | (0.007) |  |  | (0.010) | (0.002) |
| *Donor earmarked multilateral commitments (yearly, log, lagged)* | -0.055 | 0.060*** | 0.311** | 0.127*** | -0.086*** | -0.017*** |
|  | (0.101) | (0.009) | (0.109) | (0.025) | (0.019) | (0.002) |
| *Donor multilateral commitments (yearly, log, lagged)* | 0.095 | -0.029+ | 0.291 | -0.001 | 0.167*** | 0.010*** |
|  | (0.230) | (0.016) | (0.447) | (0.048) | (0.041) | (0.003) |
| *Donor bilateral commitments (yearly, log, lagged)* | -0.389 | 0.005 | -0.170 | 0.161* | -0.069 | 0.057*** |
|  | (0.261) | (0.027) | (0.388) | (0.063) | (0.050) | (0.006) |
| *Donor GDP per capita (log, lagged)* | -2.815 | 1.079*** | -7.503 | 1.558*** | 3.227*** | 0.426*** |
|  | (6.112) | (0.168) | (5.886) | (0.372) | (0.872) | (0.037) |
| *Donor democracy* | -0.553 | 1.005*** | -4.531 | -0.647 | -0.450 | 0.868*** |
|  | (2.526) | (0.279) | (4.159) | (0.664) | (0.413) | (0.050) |
| *Donor ideal-point* | -0.026 | 0.407*** | -1.675* | -0.013 | -0.523*** | -0.117*** |
|  | (0.551) | (0.057) | (0.691) | (0.163) | (0.108) | (0.011) |
| *Ideal-point distance* |  | -0.121*** |  | -0.099** |  | 0.009** |
|  |  | (0.016) |  | (0.038) |  | (0.003) |
| *Size of earmarked contribution (log)* |  |  |  |  | 0.004*** | -0.017*** |
|  |  |  |  |  | (0.001) | (0.001) |
| Observations | 14219 | 60132 | 213 | 7113 | 14192 | 59997 |
| R^2^ | 0.376 | 0.425 | 0.931 | 0.634 | 0.735 | 0.668 |
| Fixed effects: IO | Yes | Yes | Yes | Yes | Yes | Yes |
| Fixed effects: Donor | Yes | Yes | Yes | Yes | Yes | Yes |
| Fixed effects: Year | Yes | Yes | Yes | Yes | Yes | Yes |
| Fixed effects: Sector | Yes | Yes | No | No | Yes | Yes |
| Fixed effects: Recipient | Yes | Yes | No | No | Yes | Yes |
| *Clustered standard errors in parentheses; + p < 0.1, * p < 0.05, ** p < 0.01, *** p < 0.001* | | | | | | |
|  |  |  |  |  |  |  |

| **Table A9.** Alternative sampling for model 4. | | | | | | | | |
| --- | --- | --- | --- | --- | --- | --- | --- | --- |
|  | (USA size) | (G7 size) | (G12 size) | (NoUSA size) | (noG7 size) | (No G12 size) | (pre2008 size) | (post2008 size) |
| *Executive co-nationality* | -0.631** | -0.156* | -0.441*** | 0.131 | 0.046 | -0.077 | -0.008 | -0.583*** |
|  | (0.218) | (0.062) | (0.053) | (0.081) | (0.117) | (0.188) | (0.095) | (0.058) |
| *Tenure (start)* | 0.020 | 0.091** | 0.028 | 0.009 | -0.023 | 0.005 | 0.033 | -0.005 |
|  | (0.075) | (0.034) | (0.025) | (0.023) | (0.028) | (0.038) | (0.039) | (0.026) |
| *Tenure (end)* | -0.288*** | 0.017 | -0.028 | -0.033 | -0.095*** | -0.051 | 0.008 | -0.035 |
|  | (0.073) | (0.034) | (0.024) | (0.022) | (0.026) | (0.036) | (0.040) | (0.024) |
| *Number of donor-funded projects in IO (yearly, log)* | -0.979*** | -0.971*** | -0.958*** | -0.932*** | -0.951*** | -0.912*** | -0.883*** | -0.970*** |
|  | (0.037) | (0.015) | (0.011) | (0.011) | (0.014) | (0.020) | (0.020) | (0.012) |
| *Total volume of donor-earmarked funding in IO (yearly, log)* | 0.596*** | 0.748*** | 0.781*** | 0.782*** | 0.789*** | 0.775*** | 0.754*** | 0.788*** |
|  | (0.040) | (0.011) | (0.008) | (0.008) | (0.010) | (0.013) | (0.013) | (0.008) |
| *Ideal-point distance* | -0.265* | 0.038+ | -0.071*** | -0.105*** | -0.080*** | -0.046 | 0.064* | -0.119*** |
|  | (0.115) | (0.022) | (0.017) | (0.016) | (0.024) | (0.037) | (0.030) | (0.018) |
| *Co-nationality x Tenure (end)* | 0.292*** | 0.008 | -0.072 | -0.613*** | -0.630** | 0.217 | -0.153* | 0.066 |
|  | (0.082) | (0.047) | (0.044) | (0.168) | (0.219) | (0.369) | (0.076) | (0.050) |
| *Co-nationality x Tenure (start)* | -0.104 | -0.227*** | -0.216*** | -1.196*** | -1.083*** | -0.054 | -0.132+ | -0.139* |
|  | (0.090) | (0.050) | (0.047) | (0.187) | (0.224) | (0.306) | (0.076) | (0.055) |
| *Donor earmarked commitments (yearly, log, lagged)* |  | 0.005 | 0.077*** | 0.058*** | 0.069*** | 0.081*** | 0.000 | 0.158*** |
|  |  | (0.012) | (0.010) | (0.009) | (0.013) | (0.018) | (0.012) | (0.018) |
| *Donor multilateral commitments (yearly, log, lagged)* |  | 0.180*** | 0.038+ | -0.025 | -0.077*** | -0.099*** | 0.128*** | -0.059** |
|  |  | (0.032) | (0.020) | (0.017) | (0.019) | (0.028) | (0.033) | (0.019) |
| *Donor bilateral commitments (yearly, log, lagged)* |  | -0.047 | -0.081* | 0.021 | 0.014 | 0.093+ | -0.226*** | -0.071+ |
|  |  | (0.045) | (0.033) | (0.027) | (0.036) | (0.047) | (0.052) | (0.037) |
| *Donor GDP per capita (log, lagged)* |  | 0.975* | 2.100*** | 0.975*** | 1.106*** | 0.273 | -0.676 | 1.369*** |
|  |  | (0.475) | (0.311) | (0.168) | (0.203) | (0.243) | (0.546) | (0.212) |
| *Donor democracy* |  | -3.714*** | -2.442*** | 2.334*** | 2.334*** | 0.784* | 0.243 | -0.919*** |
|  |  | (0.489) | (0.376) | (0.340) | (0.366) | (0.360) | (1.457) | (0.262) |
| *Donor ideal-point* |  | -0.010 | -0.080 | 0.500*** | 0.440*** | 0.388** | 0.482*** | 0.392*** |
|  |  | (0.075) | (0.061) | (0.058) | (0.088) | (0.150) | (0.113) | (0.073) |
| Observations | 17583 | 36954 | 59621 | 56768 | 37397 | 14730 | 18233 | 56118 |
| R2 | 0.353 | 0.381 | 0.395 | 0.425 | 0.415 | 0.466 | 0.351 | 0.424 |
| Fixed effects: IO | Yes | Yes | Yes | Yes | Yes | Yes | Yes | Yes |
| Fixed effects: Donor | Yes | Yes | Yes | Yes | Yes | Yes | Yes | Yes |
| Fixed effects: Recipient | Yes | Yes | Yes | Yes | Yes | Yes | Yes | Yes |
| Fixed effects: Year | Yes | Yes | Yes | Yes | Yes | Yes | Yes | Yes |
| Fixed effects: Sector | Yes | Yes | Yes | Yes | Yes | Yes | Yes | Yes |
| *Clustered standard errors in parentheses; + p < 0.1, * p < 0.05, ** p < 0.01, *** p < 0.001* | | | | | | | | |

| **Table A10.** Alternative sampling for model 8. | | | | | | | | |
| --- | --- | --- | --- | --- | --- | --- | --- | --- |
|  | (USA volume) | (G7 volume) | (G12 volume) | (NoUSA volume) | (noG7 volume) | (No G12 volume) | (pre2008 volume) | (post2008 volume) |
| *Executive co-nationality* | -0.014 | 0.399+ | 0.488*** | 0.529*** | 0.507** | 0.147 | 0.415+ | 0.339* |
|  | (0.755) | (0.206) | (0.145) | (0.140) | (0.168) | (0.266) | (0.227) | (0.152) |
| *Tenure (start)* | 0.057 | 0.054 | -0.008 | 0.017 | 0.002 | 0.062 | 0.019 | 0.033 |
|  | (0.247) | (0.092) | (0.063) | (0.049) | (0.056) | (0.070) | (0.090) | (0.058) |
| *Tenure (end)* | 0.332 | -0.040 | -0.023 | 0.015 | 0.057 | 0.121+ | 0.007 | 0.059 |
|  | (0.282) | (0.093) | (0.064) | (0.049) | (0.057) | (0.072) | (0.096) | (0.056) |
| *Number of donor-funded projects in IO (yearly, log)* | 0.978*** | 0.926*** | 0.947*** | 0.959*** | 0.992*** | 1.044*** | 1.114*** | 0.939*** |
|  | (0.107) | (0.037) | (0.025) | (0.021) | (0.025) | (0.034) | (0.037) | (0.025) |
| *Idealpoint distance* | -0.438+ | -0.052 | -0.053 | -0.105** | -0.060 | -0.101 | -0.077 | -0.136** |
|  | (0.256) | (0.064) | (0.042) | (0.038) | (0.048) | (0.069) | (0.075) | (0.043) |
| *Co-nationality x Tenure (end)* | -0.232 | -0.136 | -0.217 | -0.224 | -0.309 | 0.090 | -0.494 | 0.066 |
|  | (0.394) | (0.318) | (0.265) | (0.292) | (0.275) | (0.374) | (0.498) | (0.233) |
| *Co-nationality x Tenure (start)* | 0.135 | -0.153 | 0.044 | -0.035 | -0.164 | -0.502 | 0.037 | -0.250 |
|  | (0.418) | (0.322) | (0.261) | (0.303) | (0.352) | (0.454) | (0.423) | (0.253) |
| *Donor earmarked commitments (yearly, log, lagged)* |  | 0.069 | 0.133*** | 0.135*** | 0.162*** | 0.122** | 0.081* | 0.202*** |
|  |  | (0.047) | (0.031) | (0.024) | (0.031) | (0.040) | (0.035) | (0.043) |
| *Donor multilateral commitments (yearly, log, lagged)* |  | -0.077 | -0.027 | -0.000 | 0.034 | 0.057 | -0.201* | 0.018 |
|  |  | (0.112) | (0.061) | (0.048) | (0.053) | (0.084) | (0.087) | (0.063) |
| *Donor bilateral commitments (yearly, log, lagged)* |  | 0.483*** | 0.096 | 0.138* | 0.063 | 0.230* | -0.022 | 0.040 |
|  |  | (0.133) | (0.089) | (0.062) | (0.077) | (0.098) | (0.118) | (0.092) |
| *Donor GDP per capita (log, lagged)* |  | 1.556 | 2.561*** | 1.566*** | 1.259** | 0.783 | 1.010 | 1.142* |
|  |  | (1.187) | (0.737) | (0.366) | (0.447) | (0.511) | (1.252) | (0.458) |
| *Donor democracy* |  | -5.133* | -1.339 | -0.767 | -0.029 | -1.029 | -10.254* | 0.020 |
|  |  | (2.033) | (1.564) | (0.662) | (0.664) | (0.702) | (4.850) | (0.676) |
| *Donor ideal-point* |  | -0.573* | -0.280 | -0.017 | 0.034 | 0.365 | -0.399 | 0.317 |
|  |  | (0.272) | (0.193) | (0.163) | (0.200) | (0.408) | (0.280) | (0.256) |
| Observations | 346 | 2209 | 4537 | 6980 | 5117 | 2789 | 2257 | 5069 |
| R^2^ | 0.832 | 0.676 | 0.625 | 0.636 | 0.628 | 0.671 | 0.616 | 0.670 |
| Fixed effects: IO | Yes | Yes | Yes | Yes | Yes | Yes | Yes | Yes |
| Fixed effects: Donor | Yes | Yes | Yes | Yes | Yes | Yes | Yes | Yes |
| Fixed effects: Year | Yes | Yes | Yes | Yes | Yes | Yes | Yes | Yes |
| *Clustered standard errors in parentheses; + p < 0.1, * p < 0.05, ** p < 0.01, *** p < 0.001* | | | | | | | | |

| **Table A11**. Alternative sampling for model 12. | | | | | | | | |
| --- | --- | --- | --- | --- | --- | --- | --- | --- |
|  | (USA stringency) | (G7 stringency) | (G12 stringency) | (NoUSA stringency) | (noG7 stringency) | (No G12 stringency) | (pre2008 stringency) | (post2008 stringency) |
| *Executive co-nationality* | -0.065** | 0.072*** | 0.054*** | 0.008 | -0.099*** | -0.060+ | 0.001 | 0.066*** |
|  | (0.023) | (0.011) | (0.010) | (0.017) | (0.022) | (0.032) | (0.023) | (0.008) |
| *Tenure (start)* | 0.063*** | 0.034*** | 0.015*** | 0.016*** | 0.015** | 0.030*** | 0.035*** | 0.007+ |
|  | (0.008) | (0.005) | (0.004) | (0.004) | (0.005) | (0.009) | (0.009) | (0.004) |
| *Tenure (end)* | 0.038** | -0.003 | 0.001 | 0.009* | 0.012* | 0.003 | 0.019* | -0.001 |
|  | (0.012) | (0.005) | (0.004) | (0.004) | (0.005) | (0.008) | (0.009) | (0.004) |
| *Size of earmarked contribution (log)* | 0.003** | -0.008*** | -0.013*** | -0.018*** | -0.018*** | -0.023*** | -0.023*** | -0.012*** |
|  | (0.001) | (0.001) | (0.001) | (0.001) | (0.001) | (0.002) | (0.002) | (0.001) |
| *Number of donor-funded projects in IO (yearly, log)* | 0.017** | 0.011*** | 0.005* | -0.012*** | -0.020*** | -0.033*** | 0.034*** | 0.002 |
|  | (0.005) | (0.003) | (0.002) | (0.002) | (0.003) | (0.005) | (0.005) | (0.002) |
| *Total volume of donor-earmarked funding in IO (yearly, log)* | 0.013* | -0.019*** | -0.009*** | -0.001 | -0.002 | -0.002 | -0.013*** | -0.008*** |
|  | (0.006) | (0.002) | (0.002) | (0.002) | (0.002) | (0.004) | (0.004) | (0.002) |
| *Ideal-point distance* | -0.055*** | 0.007 | -0.004 | 0.008** | -0.000 | -0.016* | -0.018** | 0.002 |
|  | (0.014) | (0.004) | (0.003) | (0.003) | (0.005) | (0.008) | (0.007) | (0.003) |
| *Co-nationality x Tenure (end)* | 0.025* | 0.082*** | 0.071*** | 0.023 | 0.058+ | 0.030 | 0.081*** | 0.060*** |
|  | (0.011) | (0.007) | (0.007) | (0.027) | (0.033) | (0.053) | (0.018) | (0.006) |
| *Co-nationality x Tenure (start)* | -0.084*** | -0.082*** | -0.101*** | 0.004 | 0.066* | -0.059 | 0.037* | -0.176*** |
|  | (0.011) | (0.008) | (0.007) | (0.029) | (0.033) | (0.056) | (0.015) | (0.007) |
| *Donor earmarked commitments (yearly, log, lagged)* |  | -0.024*** | -0.033*** | -0.013*** | -0.004 | 0.009+ | -0.030*** | -0.025*** |
|  |  | (0.004) | (0.002) | (0.002) | (0.003) | (0.005) | (0.003) | (0.004) |
| *Donor multilateral commitments (yearly, log, lagged)* |  | -0.052*** | 0.005 | 0.013*** | 0.026*** | 0.022*** | 0.036*** | -0.009** |
|  |  | (0.005) | (0.004) | (0.003) | (0.004) | (0.006) | (0.007) | (0.003) |
| *Donor bilateral commitments (yearly, log, lagged)* |  | 0.019+ | 0.031*** | 0.053*** | 0.061*** | 0.125*** | -0.066*** | 0.094*** |
|  |  | (0.010) | (0.006) | (0.006) | (0.007) | (0.012) | (0.011) | (0.007) |
| *Donor GDP per capita (log, lagged)* |  | 0.230* | 0.775*** | 0.419*** | 0.324*** | 0.359*** | -0.480*** | 0.448*** |
|  |  | (0.113) | (0.066) | (0.037) | (0.044) | (0.051) | (0.125) | (0.043) |
| *Donor democracy* |  | 1.428*** | 1.160*** | 0.841*** | 0.776*** | 0.914*** | -1.965*** | 0.852*** |
|  |  | (0.071) | (0.052) | (0.070) | (0.077) | (0.085) | (0.302) | (0.039) |
| *Donor ideal-point* |  | 0.148*** | -0.029* | -0.104*** | -0.098*** | -0.227*** | -0.166*** | -0.076*** |
|  |  | (0.016) | (0.012) | (0.012) | (0.020) | (0.045) | (0.027) | (0.013) |
| Observations | 17546 | 36903 | 59547 | 56643 | 37286 | 14642 | 18199 | 55990 |
| R^2^ | 0.764 | 0.678 | 0.634 | 0.665 | 0.671 | 0.711 | 0.591 | 0.699 |
| Fixed effects: IO | Yes | Yes | Yes | Yes | Yes | Yes | Yes | Yes |
| Fixed effects: Donor | Yes | Yes | Yes | Yes | Yes | Yes | Yes | Yes |
| Fixed effects: Recipient | Yes | Yes | Yes | Yes | Yes | Yes | Yes | Yes |
| Fixed effects: Year | Yes | Yes | Yes | Yes | Yes | Yes | Yes | Yes |
| Fixed effects: Sector | Yes | Yes | Yes | Yes | Yes | Yes | Yes | Yes |
| *Clustered standard errors in parentheses; + p < 0.1, * p < 0.05, ** p < 0.01, *** p < 0.001* | | | | | | | | |
